# Histamine H3 Receptor as a target for alcohol use disorder: challenging the predictability of animal models for clinical translation in drug development

**DOI:** 10.1038/s41398-026-03807-y

**Published:** 2026-01-29

**Authors:** Bernard Le Foll, Mickael Naassila, Jérôme Jeanblanc, Christian S. Hendershot, Jesus Chavarria, Thierry Calmels, Stéphane Krief, Isabelle Berrebi-Bertrand, Marilyne Uguen, David Perrin, Xavier Ligneau, Isabelle Boileau, Pablo M. Rusjan, Patricia Di Ciano, Pamela Sabioni, Marc Capet, Philippe Robert, Olivier Finance, Jeanne-Marie Lecomte, Jean Charles Schwartz

**Affiliations:** 1 Translational Addiction Research Laboratory, Campbell Family Mental Health Research Institute, Centre for Addiction and Mental Health, University of Toronto, Toronto, ON Canada; 2 Institute for Mental Health Policy Research, Centre for Addiction and Mental Health, Toronto, ON Canada; 3 Department of Family and Community Medicine, University of Toronto, Toronto, ON Canada; 4 Department of Pharmacology and Toxicology, University of Toronto, Toronto, ON Canada; 5 Institute of Medical Sciences, University of Toronto, Toronto, ON Canada; 6 Department of Psychiatry, University of Toronto, Toronto, ON Canada; 7 Campbell Family Mental Health Research Institute, Toronto, ON Canada; 8 Université de Picardie Jules Verne, Groupe de Recherche sur l’Alcool & les Pharmacodépendances, INSERM UMR1247, Amiens, France; 9 Department of Population and Public Health Sciences and Institute for Addiction Science, Keck School of Medicine, University of Southern California, Los Angeles, CA USA; 10 Department of Psychology, Western University, London, ON Canada; 11 Bioprojet-Biotech, Saint-Grégoire, France; 12 Brain Health Imaging Centre, Centre for Addiction and Mental Health, Toronto, ON Canada; 13 The Douglas Research Centre, McGill University, Montreal, QC Canada; 14 Dalla Lana School of Public Health, University of Toronto, Toronto, ON Canada; 15 Bioprojet-Pharma, Paris, France

**Keywords:** Neuroscience, Drug discovery, Addiction, Psychiatric disorders, Health sciences

## Abstract

There is an important need to advance medication development for alcohol use disorder (AUD). BP1.3656B, a highly potent and selective histamine H3 receptor inverse agonist/antagonist, has been developed. Preclinical studies revealed high affinity, good pharmacokinetic profile, good brain penetration, and favorable safety. BP1.3656B reduced alcohol drinking and alcohol-seeking behavior in rodents. Phase I studies revealed good tolerability/pharmacokinetic in humans. Positron emission tomography revealed high brain occupancy in humans. Based on this favorable profile, two trials were conducted in subjects with AUD. In non-treatment seekers, BP1.3656B had no impact on intravenous alcohol self-administration (IV-ASA). A randomized clinical trial testing three doses of BP1.3656B *versus* placebo in treatment-seekers with AUD showed no reduction of heavy drinking days. Collective results illustrate the challenges inherent to clinical translation of AUD therapies, and reinforce the use of Phase IIa human laboratory paradigms as an important tool to de-risk translation of innovative drug targets for AUD.

Alcohol Use Disorder (AUD) is highly prevalent and creates a large burden on individuals, their families and society. Current pharmacotherapies have only modest efficacy and remain underutilized. Advancing medication development for AUD is therefore a critical public health priority. In principle, the medication development pipeline begins with basic research linking specific receptors to AUD-related neurobiology, followed by testing in relevant preclinical models. Promising findings are then evaluated in proof-of-concept studies in human volunteers and, subsequently, in larger clinical trials [1]. However, it remains unclear what models should be used to develop a medication for AUD, as there are significant gaps in knowledge on effective/ineffective drugs in AUD patients due to publication bias or lack of testing [2].

Histamine has been implicated in various neuro-psychiatric disorders [3–6]. The H3 receptor (H3R) was first characterized as an autoreceptor that regulates histamine synthesis and/or release from histaminergic neurons [7]. The H3R was also identified as an hetero-receptor modulating the activity of other neurotransmitters [8], and is located post-synaptically on striatal neurons [9], making it a potential target for substance use disorder treatment [10]. Recently, a H3R inverse agonist has shown to be effective for narcolepsy/cataplexy [11], which led to FDA approval.

Preclinical studies suggest the implication of the H3R in alcohol drinking and alcohol seeking. The H3R is located in the mesolimbic system involved in addiction neurobiology [5, 10]. For example, in mice lacking H3R, alcohol consumption was reduced in a free access paradigm and conditioned place preference was attenuated [12, 13]. H3R inverse agonists/antagonists reduce lever pressing for alcohol under a fixed ratio (FR)-1 schedule of reinforcement [14] and dose-dependently decrease reinstatement of alcohol seeking (alcohol not available) [15]. Binding experiments also suggest decreased H3R levels in some brain areas in alcohol-preferring rats [16] (see also [17,18,19,20,21] for reviews on preclinical addiction models). However, to date, there is no translation of those findings into human experimental or clinical studies of AUD.

To address this gap, we have developed BP1.3656B, a novel H3R inverse-agonist. We report here for the first time its chemical structure (Fig. 1a) and the basic pharmacological studies performed demonstrating its safety profile, high affinity, and selectivity for the target. We also report its profile in preclinical alcohol studies and the results of six clinical trials (Phase 1 and 2). The first three studies tested the safety/pharmacokinetic profile after acute and sub-chronic administration of BP1.3656B in human volunteers, while the fourth study measured H3R occupancy using positron emission tomography (PET). Finally, a Phase 2a human laboratory trial tested the impact of BP1.3656B of intravenous alcohol self-administration (IV-ASA) in non-treatment-seeking adults with AUD, and a Phase 2 trial tested the impact of various doses on alcohol drinking in a multi-center randomized clinical trial involving treatment-seeking patients.

**Fig. 1:**
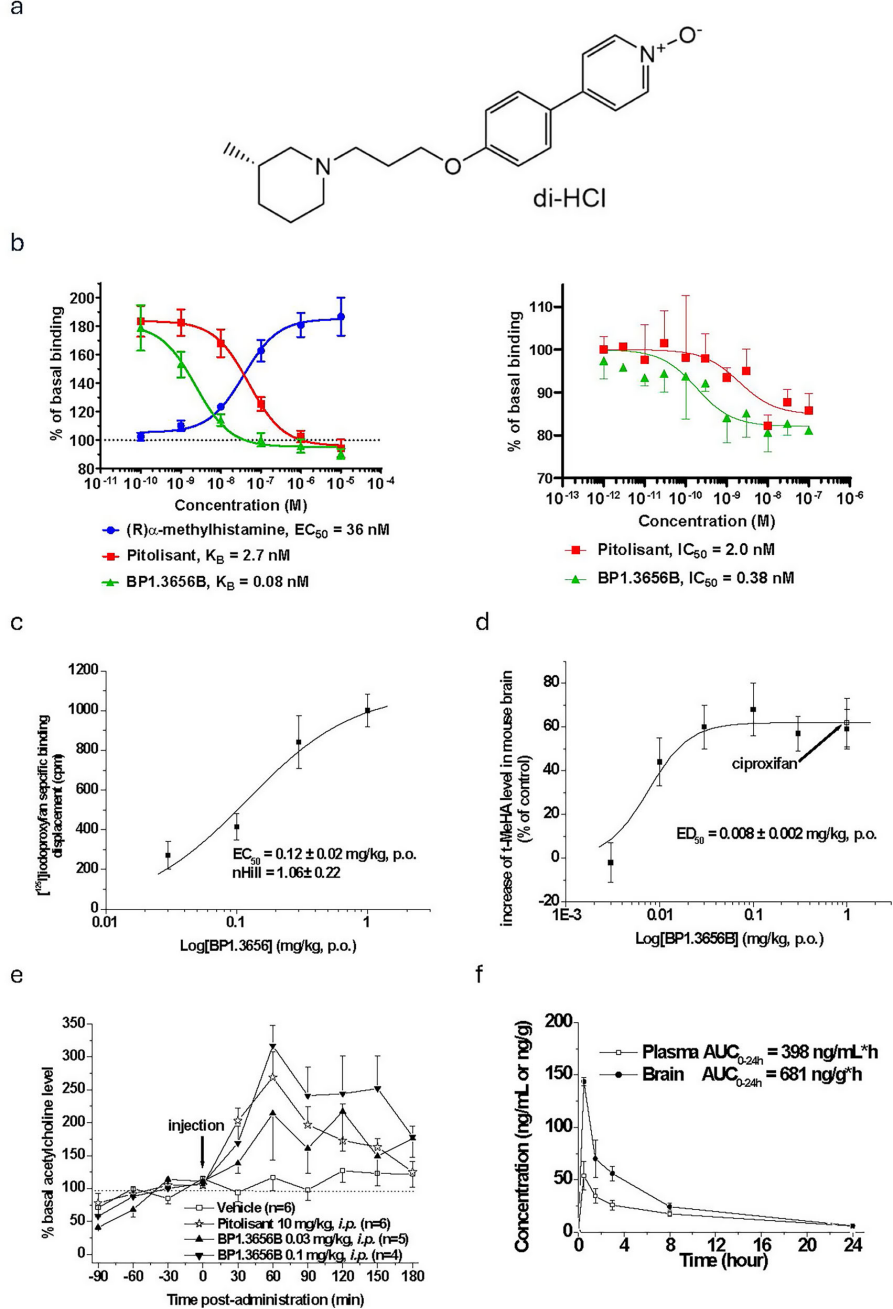
Structure and in vitro and in vivo pharmacology of BP1.3656B. **a** Structure of BP1.3656B, (*3S)-4-{4-[3-(3-methylpiperidin-1-yl)propoxy]phenyl}pyridine 1-oxide, dihydrochloride* **b** [^35^S]-GTPγS binding to membranes of HEK-293 cells stably expressing the hH3R. Reversal by pitolisant and BP1.3656B of the (R)α-methyl-histamine-induced increase in [^35^S]-GTPγS binding (left). Inhibition by pitolisant and BP1.3656B of the [^35^S]-GTPγS binding (right). **c** Reversal by pitolisant and BP1.3656B of the histamine-induced inhibition of cAMP production in forskolin-treated CHO-K1 cells stably expressing the hH3R (left) and of the histamine-induced calcium release in HEK-293 cells stably expressing the hH3R. **d** Reversal by pitolisant and BP1.3656B of the histamine-induced calcium release in HEK-293 cells stably expressing the hH3R. **e** In vivo interaction of BP1.3656A with central H3 receptors in male OF1 mice when given by oral route (example of one typical experiment with mean ± s.e.m. of at least 5 mice for each point). **f** Effect of BP1.3656B on t-MeHA level in the brain of male OF1 mice. Vehicle, BP1.3656B (0.003, 0.01, 0.03, 0.1, 0.3 and 1 mg/kg) or ciproxifan (1 mg/kg) were administered p.o. 90 min before sacrifice. t-MeHA levels are expressed in percent increase as compared to levels in control mice (227 ± 7 ng/g). Mean ± s.e.m. of 12–18 mice. **g** Effects of BP1.3656B on extracellular acetylcholine in the hippocampus of male Wistar rats. Rats received intraperitoneally vehicle, pitolisant (10 mg/kg) or BP1.3656B (0.03, 0.1, 0.3 and 1 mg/kg) and acetylcholine was determined by HPLC coupled with an electrochemical detection in brain microdialysate 30-minute samples. Mean ± s.e.m. of 4–6 rats. **h** Pharmacokinetics of BP1.3656B in male Swiss mice (1 mg/kg, p.o.). Mean ± s.e.m. of 3 mice.

## Results

### In vitro and pharmacokinetic profile of BP1.3656B in rodents

The nonclinical profile of BP1.3656B was investigated using in vitro assays to establish its affinity and functional activity on the histamine H3 receptor (H3R, see Table S01a, S01b). BP1.3656B demonstrates high affinity and potent inverse agonist activity at the human H3R, with strong selectivity over other histamine receptor subtypes and a broad panel of targets, supporting its profile as a highly selective and functionally active H3R antagonist with minimal off-target or cardiotoxic liability (see in vitro results in Extended Data). Detailed in vitro assay and pharmacokinetic results of BP1.3656B in rodents are presented in the Supplementary Material.

### Effect of BP1.3656B on alcohol self-administration, extinction, and alcohol relapse in non-dependent and dependent rats

**Fig. 2:**
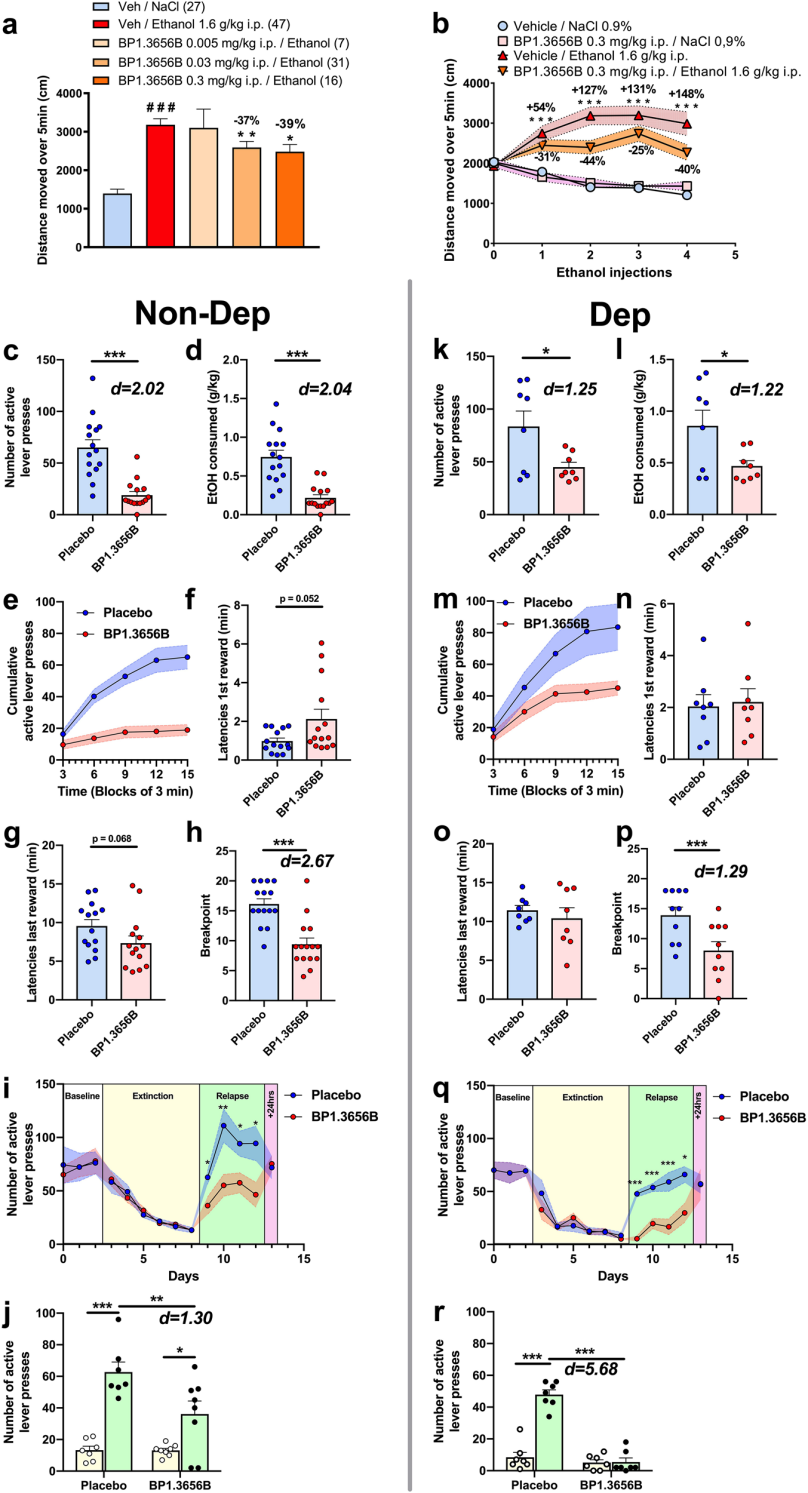
Effect of BP1.3656B in preclinical alcohol-related rodent models. **a** Effects of BP1.3656B on total distance moved over 5 min after acute ethanol injection in female DBA/2J mice. Vehicle or BP1.3656B (0.005, 0.03 or 0.3 mg/kg) were administered i.p. 30 min before acute ethanol injection (1.6 g/kg, i.p.). Mean ± s.e.m. of 7–47 mice. Statistics: one-way ANOVA followed by a PLSD Dunnett’s test: F_(4,123)_ = 16.98, *p* < 0.0001 and ### *p* < 0.001 *versus* vehicle / NaCl group, * *p* < 0.05, ** *p* < 0.001 *versus* vehicle / ethanol group. **b** Effects of BP1.3656B on acquisition of behavioural locomotor sensitization to ethanol in female DBA/2J mice. Vehicle or BP1.3656B (0.3 mg/kg) were administered i.p. 30 min before each daily ethanol injection (1.6 g/kg, i.p.). Mean ± s.e.m. of 27-31 mice. Statistics: two-way ANOVA with F_(9, 348)_ = 3.821 and *p* = 0.0001 followed by a Fisher’s LSD test: *** *p* < 0.001 *versus* vehicle / NaCl group, ## *p* < 0.01 and # *p* < 0.05 *versus* vehicle / ethanol group. **c** to **r**, Alcohol self-administration and motivation are reduced by BP1.3656B (0.3 mg/kg, i.p.) in both dependent (right) and non-dependent (left) rats while relapse is totally blocked only in dependent rats. Effect of BP1.3656B on the number of active lever presses **c,** **k**; on the amount of alcohol consumed **d,** **l**; on the motivation (breakpoint) **e,** **m**; on the cumulative active lever presses **f,** **n**; on the latency for the 1^st^ reward and last reward **g,** **h,** **o,** **p**; Effect of sub-chronic (4 consecutive days) BP1.3656B administration on the relapse after extinction **i,** **q**; and Effect of acute BP1.3656B administration on the relapse after extinction **j,** **r**. Results are expressed as mean ± s.e.m. Non-dependent rats: **c, d, e, f, g, h**: n = 15 within subjects; **i, j**: Placebo n = 7; BP1.3656B n = 8, between subjects design. Dependent rats: **k, l, m, n, o, p**: n = 8, between subject’s design; **q,** **r**: n = 8 between subjects. Statistical analysis: **c, d, f, g, h, k, l, n, o, p**: Student’s *t*-test; **i, j, q, r**: two-way ANOVA RM followed by Tukey. * *p* < 0.05, ** *p* < 0.01, *** *p* < 0.001 *versus* respective placebo group. When significant difference, Cohen’s d value for the effect size.

In non-dependent rats, baseline levels of consumption were about 0.73 g/kg/15 min (min: 0.38 g/kg/15 min – max: 1.1 g/kg/15 min). BP1.3656B significantly decreased alcohol self-administration in the non-dependent rats with a drop of 70% in the number of active lever presses (Fig. 2c) and of 73% in the levels of alcohol consumed (Fig. 2d). The cumulative presses showed that rats never reached the same rate of pressing than after the injection of the placebo (Fig. 2e). The drinking pattern indicates that the initiation of the consummatory episode (latency to the 1^st^ reward) is postponed after the injection of BP1.3656B as compared to Placebo (*p* = 0.052, Fig. 2f). BP1.3656B appeared to induce early termination of drinking episodes, as indicated by reduced latency to the last reward (*p* = 0.068; Fig. 2g), with no effect on self-administration observed the following day (data not shown). The breakpoint (*i.e*., the maximum effort to obtain a single dose of alcohol) is reduced (−43%) after BP1.3656B injection (Fig. 2h). Extinction of operant behavior was unaffected by any dose (Fig. 2i). The administration of BP1.3656B significantly reduced alcohol relapse (alcohol available) after 6 extinction sessions (44% reduction at the first day), as compared to Placebo (Fig. 2i and j). It is noteworthy the effect of BP1.3656B is present all along the alcohol relapse sessions until its administration is stopped (5^th^ day of testing). The last session of extinction and the 1^st^ sessions of alcohol relapse are depicted in Fig. 2j.

In dependent rats, the alcohol consumption observed without injection after the 10 weeks of inhalation was 0.90 g/kg/15 min, whereas it was around 0.4 g/kg/15 min before inhalation. BP1.3656B induced a 46% decrease in active lever presses (Fig. 2k) and of 55% of the amount of alcohol consumed (Fig. 2l). The cumulative presses shows that the rats injected with BP1.3656B reduced their rate of pressing after reaching 45 presses in about 9 min, whereas the placebo group continue to press along the 15-minutes session (Fig. 2n). The initiation and the termination of the consummatory episode (latencies to the 1^st^ and last reward respectively) are unaltered by BP1.3656B (Fig. 2o, p). Similarly to the non-dependent rats, BP1.3656B has no more effect on self-administration the day after the administration (data not shown). BP1.3656B induced a 44% decrease in motivation to consume alcohol (breakpoint) (Fig. 2m). None of the treatments altered the extinction when injected 15 h prior to the extinction session (Fig. 2q). On the other hand, BP1.3656B totally blocked the alcohol relapse at the 1^st^ injection (Fig. 2q, r). It is noteworthy the effect of BP1.3656B is present all along the sessions of alcohol relapse as in non-dependent animals (Fig. 2r).

### Effect of BP1.3656B on alcohol-induced locomotor activity in mice

In female DBA/2J mice, acute alcohol (1.6 g/kg, i.p.) elicited a significant 2.3-fold increase in locomotor activity (3182 ± 157 cm compared to 1397 ± 112 cm in saline-treated mice, *p* < 0.001) when looking to the total distance moved over 5 min post dosing. In mice pretreated with BP1.3656B 30 min before the alcohol injection, significant reductions of 37% and 39% (2529 ± 157 cm and 2483 ± 185 cm, *p* < 0.01 and *p* < 0.05 *versus* 3182 ± 157 in corresponding vehicle/alcohol treated mice) in the alcohol-induced hyperlocomotion were recorded with the doses of 0.03 and 0.3 mg/kg, i.p., respectively (see Fig. 2a).

When given daily over 4 consecutive days at the dose of 0.3 mg/kg, i.p., BP1.3656B had no significant effect on locomotor activity (see Fig. 2b). When given daily over 4 consecutive days at the dose of 1.6 g/kg, i.p., alcohol elicited a significant increase in the total distance moved over 5 min post alcohol dosing with 2742 ± 185 cm *versus* 1784 ± 80 cm in control saline (+ 54%, *p* < 0.001) on day 1 which became more marked over the 4 days (*i.e*., + 127%, + 131 and + 148%, *p* < 0.001 on day 2, 3 and 4, respectively). In mice pre-treated with BP1.3656B 30 min before the daily alcohol injections, reductions in the alcohol-induced hyperlocomotion were recorded as compared to corresponding vehicle-treated mice with reductions of 31%, 44%, 25% and 40% on the four successive days (*p* < 0.01 *versus* vehicle-treated alcoholised mice on days 2 and *p* < 0.05 on day 4) (see Fig. 2b).

In addition, when BP1.3656B (0.3 mg/kg, i.p.) was given 30 min before alcohol (1.6 g/kg, i.p.), alcohol had no significant effect on BP1.3656 plasma exposure when measured 10- and 30-minutes post alcohol dosing and BP1.3656B (0.3 mg/kg, i.p.) also had no significant effect on alcohol plasma exposure at these time points when compared to corresponding sessions in animals receiving BP1.3656B or alcohol alone, respectively (see Table S02).

### Effect of BP1.3656B on the 10% alcohol consumption in the drinking in the dark test in mice

We then measured the consumption of alcohol in male C57BL/6J mice in a limited access binge drinking paradigm, a stable alcohol consumption of 0.94 ± 0.03 mL over 2 h was reached representing twice the water intake (0.55 ± 0.06 mL). BP1.3656B at 0.3 mg/kg, i.p., 30 min before the test significantly decreased the alcohol binge intake by 25% (see Fig. S07).

### Effect of BP1.3656B on anxiety 24 h after alcohol withdrawal following a 24 to 29-day two-bottle free choice drinking in mice

10% alcohol solution (after an initial 1-week habituation to a 5% alcohol solution), mice drank approximately 2.5 times more ethanol 10% volume than water indicating a higher preference for ethanol over water (data not shown). Twenty-four hours after alcohol withdrawal, the time spent in the open arms in the elevated plus maze test was significantly reduced by 48% in alcohol-deprived mice as compared to the time in corresponding non-deprived mice (43.9 ± 5.6 s *versus* 84.4 ± 14.9 s, respectively), whereas the time spent in the open arm in these non-deprived mice was similar to the one recorded in control mice never exposed to alcohol (64.5 ± 5.7 s). A pretreatment by BP1.3656B (0.3 mg/kg, i.p.) of alcohol-deprived mice elicited a significant increase in the time spent in the open arms (69.8 ± 6.6 s *versus* 43.9 ± 5.6 s in vehicle-treated mice, *p* < 0.01) with an almost full normalization of this parameter (see Fig. S08).

### Phase 1 clinical studies

In the first Phase I clinical study (P08-04), 45 subjects received single oral doses of BP1.3656B ranging from 1 to 150 μg (48 observations, with 3 subjects participating in two study parts three months apart) to assess pharmacokinetics (PK), safety, and pharmacodynamics (PD). PK data suggested a dose-response relationship: Cmax and AUC (AUC₀–t and AUC₀–∞) were proportional between 10 and 150 μg. PK parameters were not calculated for 1 and 3 μg due to bioanalytical levels below the quantification limit (10 pg/mL). Tmax and t½ remained consistent ( ~ 3.5 h and 15 h, respectively) across 30–150 μg doses. Statistical analysis (ANOVA) showed no dose effect on pharmacodynamic parameters. All doses were well tolerated, with no serious adverse events. Reported adverse events aligned with the H3 receptor antagonist mechanism. PD effects included reduced sleep duration and increased sleep onset latency (data not shown).

In the second Phase I study (P10-02), BP1.3656B was administered daily at 60 and 90 μg for 10 days in 12 healthy volunteers to evaluate PK, safety, and PD effects via polysomnography and EEG. Cmax was reached 2.5–3.5 h post-dose on Days 1 and 10. After repeated dosing, Cmax increased by 42% (60 μg) and 101% (90 μg), with corresponding AUC₀–₂₄h increases of 73% and 113%, respectively. Mean t½ on Day 10 was approximately 33 h, longer than the ~16 h observed in P08-04. Dose effect (placebo, 60 μg, 90 μg and 120 μg or 30 μg) analysis for Polysomnography & Cognitive and awakening tests: Repeated Measures ANOVA (including fixed effect for time and random effect for subject), completed, in case of a significant dose effect, by a 2 by 2 comparison between dose groups (Tukey or Dunnett’s test with placebo dose as reference). No significant differences were found in EEG, cognitive, awakening tests, or polysomnography. The main safety concern was mild to moderate insomnia reported in all subjects at 90 μg from Day 2 onwards. (Table S03).

The third Phase I study (P14-03) assessed safety, tolerability, PK, and cardiovascular effects of chronic daily dosing (30, 60, and 90 μg) over 21 days in healthy male and female subjects. QTcF effects were assessed using moxifloxacin as a positive control and food effect as assay sensitivity. In males, median Tmax ranged from 2.5 to 4 h on Days 1 and 21. After repeated dosing, t½ was 70 h (males) and 133 h (females) at 60 μg, and 124 h in males at 90 μg. Steady state appeared by Days 10–11. Accumulation was observed in Cmax and AUC₀–₂₄h. In males, AUC₀–₂₄h accumulation was ~100%, while Cmax accumulation varied (44% at 30 μg, 74% at 60 μg, 62% at 90 μg). In females, Cmax and AUC₀–₂₄h accumulation were higher (105% and 169%, respectively). Change from baseline in QTcF were analyzed per time points using ANOVA models including treatment as factor and subject as random effect. ΔQTcF and their 90% confidence intervals were obtained by time points. QTcF analyses showed no notable changes from baseline (data not shown).

### PET studies in humans after acute and chronic administration

**Fig. 3:**
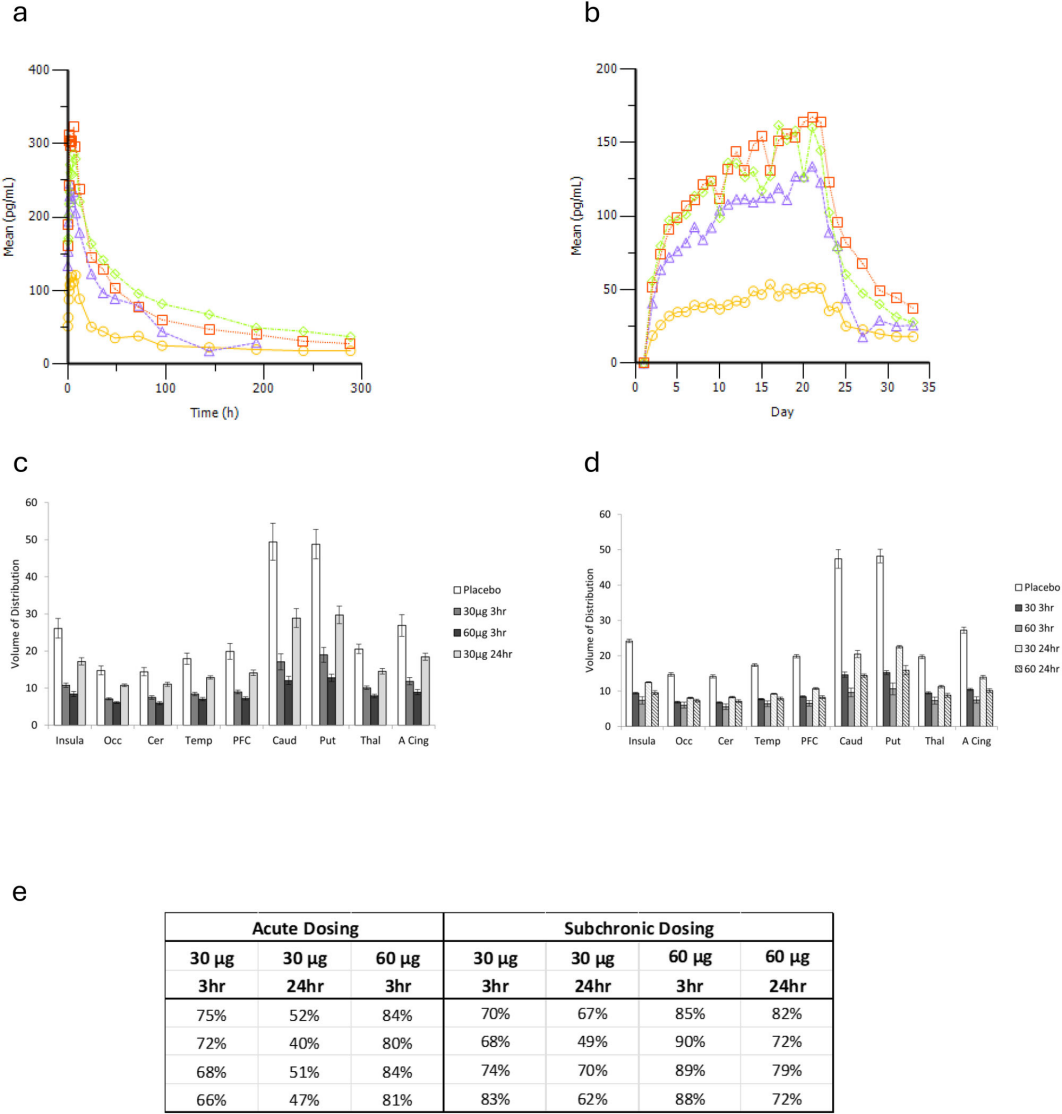
BP1.3656B: Phase I clinical studies. **Repeated administration of BP1.3656B for 21 days (P14-03 study code). a** Mean BP1.3656 serum concentrations *versus* time profiles by dose level on Day 21. (natural scale). Yellow line: Dose level 30 μg once a day in male subjects; Purple line: Dose level 60 μg once a day in male subjects; Red line: Dose level 90 μg once a day in male subjects; Green line: Dose level 60 μg once a day in female subjects **b**, Mean BP1.3656 serum concentrations *versus* time profiles by dose level at pre-dose (trough level) from Day 1 to end of study (natural scale). Yellow line: Dose level 30 μg once a day in male subjects; Purple line: Dose level 60 μg once a day in male subjects; Red line: Dose level 90 μg once a day in male subjects; Green line: Dose level 60 μg once a day in female subjects **PET studies in humans (P14-08 study code) c,** **Acute dosing**. Mean ± s.e.m [^11^C]-GSK189254 total volume of distribution (V_T_) in regions of interest following placebo (open symbols) or after single acute doses of BP1.3656B. n = 4. Participants were scanned 3 h after either 30 µg (grey bars; 30 3 hr) or 60 µg (dark grey bars; 60 3 hr) or 24 h after 30 µg (light grey bars; 30 24 hr). BP1.3656B was significantly different from placebo (*p* < 0.05) in all regions of interest for all pre-treatments. Occ: occipital cortex; Cer: cerebellum; Temp: temporal lobe; PFC: prefrontal cortex; Caud: caudate; Put: putamen; Thal: thalamus; A. Cing: anterior cingulate cortex. **d** **Subchronic dosing**. Mean ± s.e.m total volume of distribution (V_T_) in regions of interest during a PET scan with [^11^C]-GSK189254 following placebo (open symbols) or subchronic doses of BP1.3656B administered once daily for approximately 10 days. n = 4. Participants were scanned either 3 h or 24 h after either 30 µg (dark grey bars and light stippled bars, respectively; 30 3 hr, 30 24 hr) or 60 µg (3 h: light grey bars, 60 3 hr; 24 hr: dark stippled bars, 60 24 hr). BP1.3656B was significantly different from placebo (*p* < 0.05) in all regions of interest for all pre-treatments. Occ: occipital lobe; Cer: cerebellum; Temp: temporal lobe; PFC: prefrontal cortex; Caud: caudate; Put: putamen; Thal: thalamus; A. Cing: anterior cingulate cortex. **e** **Apparent receptor occupancy** (Left: Acute dosing; Right: Subchronic dosing) provided for individual participants, with mean and s.e.m.

Acute administration of 30 µg or 60 µg of BP1.3656B 3 h prior to PET scanning with [^11^C]-GSK189254 resulted in a reduction of the total distribution volume (V_T_) values (vehicle *versus* BP1.3656B) for each brain region of interest (see Fig. 3c) and corresponding apparent brain occupancy of the H3R of 70% and 82%, respectively (See Fig. 3e) Plasma BP1.3656 was 2-fold higher following 60 µg than 30 µg. When measured 24 h after 30 µg of BP1.3656B, plasma levels of BP1.3656 were low and H3R occupancy was also relatively low, at 48%.

Sub chronic administration of either 30 µg or 60 µg of BP1.3656B for 5–10 days prior to scanning with [^11^C]-GSK189254 revealed V_T_ values (vehicle *versus* BP1.3656B) for each brain region of interest (see Fig. 3d) and corresponding apparent occupancy of 74% and 88%, respectively, when scanned with [^11^C]-GSK189254 3 h after administration of BP1.3656B (see Fig. 3e). When scanned with [^11^]-GSK189254 24 h after BP1.3656B, occupancy was reduced, at 62% and 76%, for the 30 µg and 60 µg doses, respectively (see Fig. 3e). Plasma BP1.3656 was different between 30 µg and 60 µg and was lower when measured 24 h after the last dose (Data not shown).

### Effects on intravenous alcohol self-administration in subjects with AUD

**Fig. 4:**
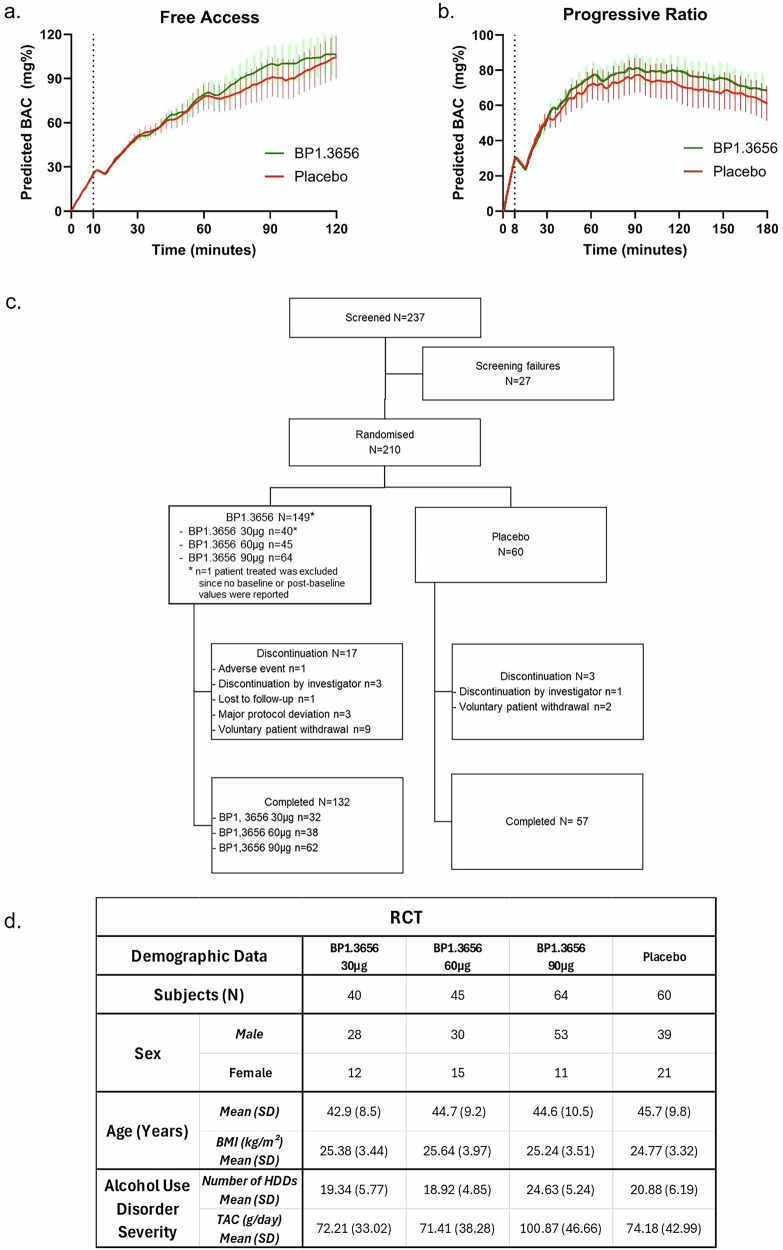
Clinical trials in subjects with AUD (Self-administration and RCT presentation). **a** Effects of BP1.3656B and placebo on intravenous alcohol self-administration (IV-ASA) during a free access trial among individuals with AUD. Random-intercept multilevel models (MLM) of area under the curve (AUC) of alcohol exposure showed no significant effect of medication type on alcohol exposure (*B[SE]* = 296.66 [1019.66], *p* = 0.771). Covariates included session number, medication distribution order, and sex. BAC=blood alcohol concentration =30 mg% alcohol prime dose. **b** Effects of BP1.3656B and placebo on IV-ASA during a progressive ratio trial among individuals with AUD. Random-intercept multilevel models of AUC of alcohol exposure showed no significant effect of medication on alcohol exposure (*B[SE]* = 774.37[1351.06], *p* = 0.567). Covariates included session number, medication distribution order, and sex. BAC=blood alcohol concentration =30 mg% alcohol prime dose. **c** Disposition of patients in the RCT. **d** Demography and baseline disease characteristics in the RCT.

A total of 36 non-treatment-seeking participants were randomized to medication or placebo in the Phase 2a human laboratory trial. After accounting for discontinuations, missed sessions, and session exclusions (*e.g*., equipment failure), 16 and 14 participants completed FA and/or PR sessions, respectively, yielding a final sample of 21 participants (43% male, mean age: 40.79 years). Alcohol exposure under free-access (FA) and progressive ratio (PR) conditions was indexed by total area under the blood alcohol concentration curves (AUCs; see Fig. 4a, b), estimated from model-projected BAC values at 30-second intervals. Random-intercept multilevel models (controlling for sex, medication order, and session order) showed no treatment effects on alcohol self-administration in either session (ns > 0.05), indicating that sub-acute BP1.3656B treatment did not alter alcohol motivation or self-administration in adults with AUD. BP1.3656B was well tolerated, with no treatment-related adverse events or discontinuations.

### Randomized clinical trial testing BP1.3656B to reduce alcohol consumption in subjects with AUD

A total of 210 patients were enrolled (Fig. 4c), with 209 included in the Full Analysis Set (FAS). Baseline demographic and clinical characteristics were well balanced across treatment groups (Table S04). Most participants were male (71.8%), with a mean age of 44.6 ± 9.6 years; 53.1% were aged 35–49 (Fig. 4d). The safety population included all 210 randomized patients who received at least one dose: 43 received BP1.3656B 30 μg, 43 received 60 μg, 64 received 90 μg, and 60 received placebo. BP1.3656B demonstrated a favorable safety profile (Table 1).

**Table 1:** Safety data of Phase 2 studies.

Safety Data								
	BP1.3656B 30 μg (N = 43)		BP1.3656B 60 μg (N = 43)		BP1.3656B 90 μg (N = 64)		Placebo	
							(N = 60)	
TEAEs	*nAEs*	*n (%) participants*	*nAEs*	*n (%) participants*	*nAEs*	*n (%) participants*	*nAEs*	*n (%) participants*
Any TEAE	14	10 (23.3%)	25	12 (27.9%)	48	18 (28.1%)	31	20 (33.3%)
Any Serious TEAE	0	0	0	0	2	1 (1.6%)	2	2 (3.3%)
**Severity**								
Mild	11	8 (18.6%)	19	10 (23.3%)	38	15 (23.4%)	22	16 (26.7%)
Moderate	3	2 (4.7%)	5	3 (7.0%)	10	8 (12.5%)	7	5 (8.3%)
Severe	0	0	1	1 (2.3%)	0	0	2	2 (3.3%)
Any drug-related TEAE	8	5 (11.6%)	15	7 (16.3%)	35	11 (17.2%)	10	10 (16.7%)
Any drug-related serious TEAE	0	0	0	0	0	0	0	0
Any TEAE leading to discontinuation	1	1 (2.3%)	7	1 (2.3%)	0	0	0	0
**Any TEAEs by System Organ Class**								
Psychiatric disorders	0	0	6	4 (9.3%)	18	8 (12.5%)	6	5 (8.3%)
Nervous system disorders	3	2 (4.7%)	4	4 (9.3%)	7	5 (7.8%)	2	2 (3.3%)
Gastrointestinal disorders	0	0	2	2 (4.7%)	7	6 (9.4%)	6	6 (10.0%)
Investigations	5	3 (7.0%)	4	3 (7.0%)	3	3 (4.7%)	0	0
General disorders and administration site conditions	2	2 (4.7%)	1	1 (2.3%)	2	2 (3.1%)	2	2 (3.3%)
Metabolism and nutrition disorders	0	0	2	2 (4.7%)	4	3 (4.7%)	1	1 (1.7%)
Musculoskeletal and connective tissue disorders	0	0	2	2 (4.7%)	2	2 (3.1%)	3	3 (5.0%)
Infections and infestations	1	1 (2.3%)	1	1 (2.3%)	1	1 (1.6%)	2	2 (3.3%)
Injury, poisoning and procedural complications	0	0	0	0	2	1 (1.6%)	3	2 (3.3%)
Respiratory, thoracic and mediastinal disorders	2	2 (4.7%)	2	1 (2.3%)	0	0	0	0
Blood and lymphatic system disorders	0	0	0	0	0	0	2	2 (3.3%)
Cardiac disorders	0	0	0	0	1	1 (1.6%)	1	1 (1.7%)
Ear and labyrinth disorders	0	0	1	1 (2.3%)	0	0	1	1 (1.7%)
Vascular disorders	1	1 (2.3%)	0	0	1	1 (1.6%)	0	0
Renal and urinary disorders	0	0	0	0	0	0	1	1 (1.7%)
Skin and subcutaneous tissue disorders	0	0	0	0	0	0	1	1 (1.7%)

### Primary outcome

For continuous endpoints on TLFB parameters, the significance of the dose effect was carried out by an analysis of covariance (ANCOVA) on the value at final time (at end of the 12-week double-blind randomised study treatment period) in adjusting for baseline value and treatment considered as a fixed factor. In addition, for the main endpoint, the centre effect was added as a random factor.

**Fig. 5:**
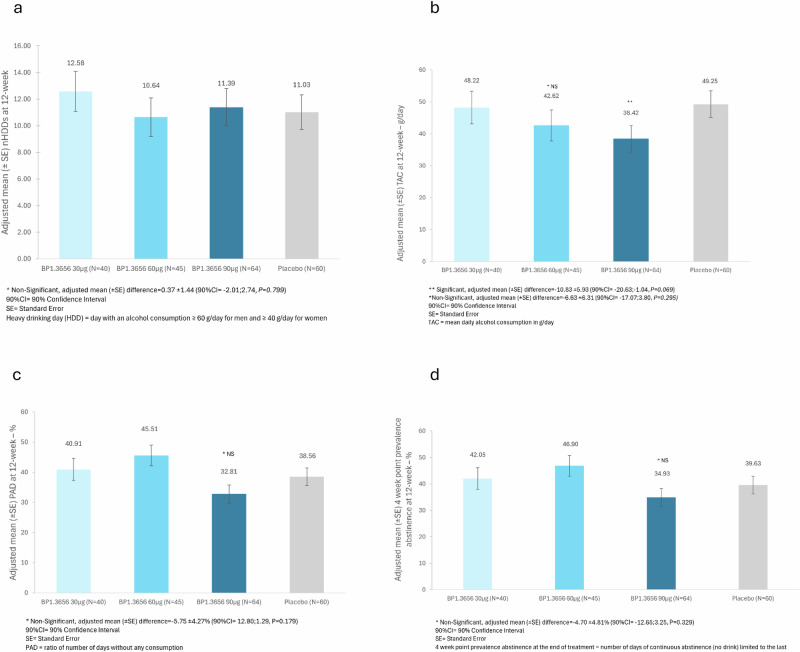
Results of the RCT in subjects with AUD. **a Effect of BP1.3656B in reducing the number of monthly Heavy Drinking Days (nHDDs) during 12-week double-blind period (primary endpoint)**. Adjusted mean (±SE) nHDDs at 12-week. Statistics: analysis of covariance (ANCOVA) on the value at 12-week with imputation, adjusting for baseline value and with treatment considered as a fixed factor and the centre effect added as a random factor. A step-down strategy was performed to assess the significance of doses, which were tested starting with the highest and considering the lowest based on the significance effect of the previous dose. **b Effect of BP1.3656B on the Total daily Alcohol Consumption (TAC) from baseline to the end of treatment (secondary endpoint)**. Adjusted mean (± SE) TAC at 12-week. Statistics: analysis of covariance (ANCOVA) on the value at 12-week with imputation, adjusting for baseline value and with treatment considered as a fixed factor. A step-down strategy was performed to assess the significance of doses, which were tested starting with the highest and considering the lowest based on the significance effect of the previous dose. **c Effect of BP1.3656B on the Percent of Abstinent Days (PAD) during 12-week double-blind period (secondary endpoint)**. Adjusted mean (± SE) PAD at 12-week. Statistics: analysis of covariance (ANCOVA) on the value at 12-week with imputation, adjusting for baseline value and with treatment considered as a fixed factor. A step-down strategy was performed to assess the significance of doses, which were tested starting with the highest and considering the lowest based on the significance effect of the previous dose. **d Effect of BP1.3656B on the 4-week point prevalence abstinence (PPA) at the end of treatment (secondary endpoint)**. Adjusted mean (±SE) 4 week point prevalence abstinence at 12-week. Statistics: analysis of covariance (ANCOVA) on the value at 12-week with data missing imputation, adjusting for baseline value and with treatment considered as a fixed factor. A step-down strategy was performed to assess the significance of doses, which were tested starting with the highest and considering the lowest based on the significance effect of the previous dose.

At baseline, mean (±SD) nHDDs in the FAS population were: 19.34 ± 5.77 (BP1.3656B 30 μg), 18.92 ± 4.85 (60 μg), 24.63 ± 5.24 (90 μg), and 20.88 ± 6.19 (placebo; Fig. 4d). After 12 weeks of double-blind treatment, nHDDs decreased across all groups: 9.49 ± 9.69 (30 μg), 7.70 ± 8.51 (60 μg), 11.82 ± 10.21 (90 μg), and 10.20 ± 9.48 (placebo; Fig. 5a).

Mean (±SD) reductions in nHDDs were: −9.85 ± 6.20 (30 μg), −11.22 ± 7.22 (60 μg), −12.80 ± 8.57 (90 μg), and −10.68 ± 8.39 (placebo). A step-down analysis tested dose significance starting from the highest dose. Adjusted mean (± SE) nHDDs at 12 weeks were: 12.58 ± 1.51 (30 μg), 10.64 ± 1.46 (60 μg), 11.39 ± 1.40 (90 μg), and 11.03 ± 1.31 (placebo). No statistically significant difference was observed between the 90 μg group and placebo.

### Secondary outcomes

#### Total daily alcohol consumption (TAC)

At baseline, mean (± SD) TAC (g/day) in the FAS population was 72.21 ± 32.02 (BP1.3656B 30 μg), 71.41 ± 38.28 (60 μg), 100.87 ± 46.66 (90 μg), and 74.18 ± 42.99 (placebo; Fig. 4d). TAC decreased across all groups over the 12-week period. Placebo data were pooled for exploratory analysis, though baseline severity appeared higher in placebo part 2; results should therefore be interpreted with caution.

At 12 weeks, adjusted mean (± SE) TAC was 48.22 ± 5.08 (30 μg), 42.62 ± 4.80 (60 μg), 38.42 ± 4.14 (90 μg), and 49.25 ± 4.15 g/day (placebo; Fig. 5b). No significant difference was found for the 60 μg group *versus* placebo (mean difference: 6.63 ± 6.31; 90% CI: 17.07 to −3.80; *p* = 0.295).

#### Controlled continuous drinking

A small percent of patients reported no HDDs during the 12-week period: 10.0% (BP1.3656 30 μg), 13.3% (BP1.3656B 60 μg), 4.7% (BP1.3656B 90 μg), and 13.3% (placebo). There was no statistical treatment difference according to multivariate logistic regression. Odds-ratio [90% CI] = 0.60 [0.18; 2.07]; *p* = 0.501.

#### Percent of abstinent days (PAD)

The adjusted mean (±SE) PAD at 12-week was 40.91 ± 3.65% in the BP1.3656B 30 μg group, 45.51 ± 3.44% in the BP1.3656B 60 μg group, 32.81 ± 3.00% in the BP1.3656B 90 μg group, and 38.56 ± 2.95% in the placebo group (Fig. 5c). The PAD increased from baseline to 12-week in all treatment groups. No statistically significant treatment differences were observed.

#### Continuous abstinence duration

A small percent of patients reported continuous abstinence: 5.0% (BP1.3656B 30 μg), 4.4% (BP1.3656B 60 μg), 1.6% (BP1.3656B 90 μg), and 3.3% (placebo). There was no statistical treatment difference according to multivariate logistic regression. Odds-ratio [90% CI] = 1.42 [0.16; 12.52]; *p* = 0.790.

#### 4-week point prevalence abstinence (PPA)

The adjusted mean (± SE) 4-week point prevalence abstinence to 12-week was 42.05 ± 4.12% in the BP1.3656B 30 μg group, 46.90 ± 3.91% in the BP1.3656B 60 μg group, 34.93 ± 3.39% in the BP1.3656B 90 μg group, and 39.63 ± 3.33% in the placebo group (Fig. 5d). The PPA increased from baseline to the 4-week point evaluation in all treatment groups. No statistically significant treatment differences were observed.

#### Alcohol biomarkers

The adjusted mean (±SE) ALAT level at 12-week was 1.30 ± 0.04 in the BP1.3656B 30 μg group, 1.35 ± 0.03 in the BP1.3656B 60 μg group, 1.33 ± 0.03 in the BP1.3656B 90 μg group, and 1.39 ± 0.03 in the placebo group. There was no statistical difference between BP1.3656B 90 μg and placebo as the adjusted mean (± SE) difference was −0.06 ± 0.04 (90%CI = −0.13;0.00, *p* = 0.124).

The adjusted mean (± SE) ASAT level at 12-week was 1.37 ± 0.03 in the BP1.3656B 30 μg group, 1.43 ± 0.03 in the BP1.3656B 60 μg group, 1.38 ± 0.02 in the BP1.3656B 90 μg group, and 1.45 ± 0.03 in the placebo group. There was a statistical difference between BP1.3656B 90 μg and placebo as the adjusted mean (± SE) difference was −0.07 ± 0.04 (90%CI = −0.13; −0.01, *p* = 0.046).

The adjusted mean (± SE) CDT level at 12-week was 0.018 ± 0.003% in the BP1.3656B 30 μg group, 0.019 ± 0.002% in the BP1.3656B 60 μg group, 0.021 ± 0.002% in the BP1.3656B 90 μg group, and 0.022 ± 0.002% in the placebo group. There was no statistical difference between BP1.3656B 90 μg and placebo as the adjusted mean (± SE) difference was −0.000 ± 0.003 (90%CI = −0.005;0.004, *p* = 0.936).

#### Craving

Craving was studied by using self-administrated Obsessive Compulsive Drinking Scale (OCDS). The adjusted mean (± SE) craving total score at 12-week was 9.1 ± 1.1 in the BP1.3656B 30 μg group, 11.6 ± 1.0 in the BP1.3656B 60 μg group, 12.2 ± 0.8 in the BP1.3656B 90 μg group, and 11.3 ± 0.8 in the placebo group. Craving total score decrease from baseline to 12-week in all treatment groups. There was no statistical difference between BP1.3656B 90 μg and placebo from baseline to 12-week as the adjusted mean (± SE) difference in total score at 12-week was 0.9 ± 1.2 (90%CI = −1.0;2.8, *p* = 0.440).

#### Depression

Depression was assessed by using Beck Depression Inventory (BDI). Overall, there was an improvement in symptoms and overt behavioral manifestations of depression in all treatment groups through the 12-week treatment period, with no treatment differences. Adjusted mean difference (BP1.3656B 90 μg *versus* Placebo) (SE) = −0.7 (0.5); *p* = 0.172.

#### Sleep quality

Sleep quality was assessed by using Pittsburgh Sleep Quality Index (PSQI). The adjusted mean (± SE) on subjective sleep quality and on daytime dysfunction at 12-week seemed to be in favour of BP1.3656B 90 μg compared to placebo (*p* = 0.029 and *p* = 0.049, respectively), while there were no observed treatment differences regarding the other component summary measures of the PSQI. Adjusted mean difference (BP1.3656 90 μg *versus* Placebo) (SE) = −0.5 (0.4); *p* = 0.303. There was an improvement in sleep quality and sleep disturbance in all treatment groups through the 12-week treatment period.

#### Quality of life

Quality of life was assessed by using 12-Item Short-Form Health Survey (SF-12) scale. Overall, there was an improvement, based on the mental and physical component summary measures of the SF-12, in all treatment groups through the treatment period.

The adjusted mean (± SE) of mental component summary seemed to be larger in the BP1.3656B 90 μg group than in the placebo at 12-week at 5% one-sided (*p* = 0.040), while there were no observed treatment differences regarding the physical component summary measures of the SF-12. Adjusted mean difference (BP1.3656B 90 μg *versus* Placebo) (SE) = −1.194 (0.893); *p* = 0.183. There was a statistical difference between BP1.3656B 90 μg and placebo as the adjusted mean (± SE) difference was 2.696 ± 1.302 (90%CI = 0.544;4.849, *p* = 0.040).

## Discussion

BP1.3656B is a novel inverse agonist for the H3R. This compound has a much higher affinity compared to an existing drug (pitolisant). This profile allows to produce behavioral effects at a much lower dosage.

Consistent with previous studies implicating H3R in alcohol-related motivation, BP1.3656B showed a promising profile for AUD across multiple preclinical models. It did not affect baseline locomotion but significantly reduced alcohol-induced hyperactivity, likely reflecting reduced dopaminergic stimulation. In mice, it modestly reduced binge drinking in the drinking-in-the-dark model. In rats, BP1.3656B strongly decreased alcohol self-administration and motivation, both in the non-dependent and dependent animals. Thus, there was a clear impact on the motivation to drink alcohol across species (mice/rats) and models. BP1.3656B was able to reduce alcohol relapse, and alcohol deprivation-related anxiety, both in non-dependent and dependent rats (Fig. 2). These findings suggest BP1.3656B may attenuate alcohol’s reinforcing effects and reduce alcohol relapse, although its impact on withdrawal symptoms was not directly tested.

Based on those promising findings, the decision was made to pursue the clinical development of this drug. The good tolerability of BP1.3656B was confirmed in the subsequent studies in human volunteers. Three phase I studies indicated good tolerability after acute and chronic administration. Notably, the most common adverse events were sleep-related disturbances, consistent with the known wake-promoting effects of H3R antagonists, as observed in narcolepsy treatment.

To confirm target engagement, H3R occupancy was assessed in humans following BP1.3656B administration using [^11^C]-GSK189254 PET. Consistent with preclinical occupancy findings, in humans a significant occupancy of H3R was obtained at 30 µg or 60 µg (like occupancy following 40 mg of pitolisant [22]. We then performed phase 2 trials to determine the impact of BP1.3656B in motivation for alcohol. Based on preclinical evidence of reduced alcohol consumption and self-administration, we designed a human laboratory self-administration design in non-treatment seekers with AUD. However, no effect on alcohol motivation was observed under either reinforcement schedule. A subsequent multicenter trial found no significant effects on multiple drinking measures across a range of doses.

Our study has several strengths. It employed diverse preclinical models to assess alcohol-related motivation in both mice and rats, including non-dependent and dependent states. Phase I trials incorporated both acute and subchronic dosing, and PET imaging was used to ensure that target will be engaged in subsequent phase 2 studies. Importantly, similar and translational models were used to evaluate alcohol motivation in both preclinical and human laboratory settings. Additionally, a range of BP1.3656B doses were tested in treatment-seeking individual with AUD. These approaches align with ongoing efforts to evaluate the predictive value of preclinical models in medication development [2].

However, this study has some limitations. For example, preclinical testing was limited to rodent models and non-human primate models were not used [23]. While H3R’s role in narcolepsy is conserved across species [24–27], this may not expand to its role in alcohol-related neurobiology. In addition, we did not use alcohol-preferring rats, nor samples coming from alcohol biobank. In humans we only used the IV-ASA paradigm, although multiple laboratory paradigms exist [2, 28]. Therefore, we cannot exclude that BP1.3656B could have effects in other paradigms. The use of IV-ASA may appear less valid than oral ingestion, but has been shown to have some advantages, notably to standardize the exposure to alcohol [29]. We also opted to study non-treatment seekers, in line with NIAAA guidelines [30], though treatment-seeking individuals may respond differently.

Regarding the RCT, the primary outcome was nHDD, a measure validated by the EU and used in nalmefene approval for AUD treatment [31]. While different trial designs might yield different outcomes, no effects were observed on multiple secondary measures of alcohol consumption and abstinence. We also believe the lack of efficacy in unlikely to reflect a lack of engagement of the target, as our PET studies confirmed H3R occupancy even at the lower doses. It should be noted that we observed a reduction of drinking measures in all groups, a finding likely reflecting a potential placebo effect and the influence of engaging in a treatment to reduce drinking.

Although our Phase 2 trials yielded negative results, our findings suggest that behavioral pharmacology laboratory experiments, due to their lower cost compared to clinical trials, could be used as effective tools to de-risk the translation of basic research to humans. It should be noted that not all medications for AUD work the same way, it is therefore important to determine if the outcome measures in human subjects would be well aligned with the outcome measure in preclinical models. In our study, the use of parallel outcome measures in both settings allowed the lack of efficacy observed in human laboratory testing to signal a potential lack of translation, which could have served as an early no-go decision for the larger clinical trial. We suggest that incorporating behavioral pharmacology Phase IIa human laboratory paradigms may serve as an important tool to de-risk translation of innovative drug targets for AUD.

## Materials and methods

### [^125^I]-iodoproxyfan binding

Human embryonic kidney (HEK293) cells stably expressing the human H3 receptor (Acc# NM_007232) were collected by addition of trypsin solution (500 µg/mL trypsin/ 200 µg/mL EDTA, Lonza BE17-161E), washed and homogenized with a Polytron in ice-cold phosphate buffer (50 mM Na_2_HPO_4_/K_2_HPO_4_, pH 7.5) and centrifuged (23 000 x g, 30 min, +4 °C). Mouse or rat brain cortex were homogenized with a Polytron in the ice-cold phosphate buffer, centrifuged (140 x g, 10 min, +4 °C) and the supernatant centrifuged (23 000 x g, 30 min, +4 °C). Final pellets were resuspended in the phosphate buffer to get membrane preparations. Binding experiments were performed according to Ligneau et al. [32] with slight modification. Membranes (5–15 µg protein/incubation) were incubated for 1 h at room temperature under continuous stirring alone or together with tested compounds in increasing concentrations in a final volume of 200 µL in the presence of 25 pM of [^125^I]-iodoproxyfan. Non-specific binding was determined using 1 µM imetit, a specific H3-receptor agonist. Incubations, performed at least in triplicate, were stopped by rapid filtration on Whatman GF/B glass fibre membranes pre-soaked in 0.3% polyethyleneimine. Radioactivity trapped on filters was directly measured in a liquid scintillation counter with 50 µL of scintillation fluid using a gamma counter. For HEK-hH3R cells, the hH3 binding investigated by use of [^125^I]-iodoproxyfan gave a B_max_ = 0.6 pmol/mg protein and a Kd = 0.044 pM. No binding was detectable in wild type HEK293 cell membranes.

### [^35^S]-GTPγS binding on the H3R

HEK293 cells that expressed the human or mouse H3R (Acc #: NM_007232, NM_133849, respectively) were grown until confluence ( + 37 °C in a 95:5 air:CO_2_ atmosphere), collected by addition of trypsin solution (500 µg/mL trypsin and 200 µg/mL EDTA, Lonza, ref. BE17-161E), then centrifuged at 300 x g for 15 min at +4 °C. Pellets were resuspended in buffer I (50 mM Tris-HCl, 10 mM MgCl_2_, 140 mM NaCl, pH 7.4) that was supplemented by 1 mM phenyl methyl sulfonyl fluoride. The suspension was stirred gently and submitted to mechanical pressure exerted through a syringe with a 25–26 G needle. The cell lysate was then centrifuged at 300 g for 5 min at +4 °C to eliminate nuclei and cell debris. The resulting supernatant was then centrifuged at 48,000 g for 30 min at +4 °C. The final pellet was resuspended in buffer I. The aliquots were frozen in liquid nitrogen and stored at −80 °C until required. Protein content was measured by the Bradford method (Bradford, 1976). Membranes were thawed, diluted to a final concentration of 2.5 µg protein/180 µL/well in 96-well polystyrene microplates and incubated for 30 min at room temperature with tested compounds in increasing concentrations in buffer I that was supplemented with guanosine diphosphate (10 µM). Labelled [^35^S]-guanosine 5’-O-[gamma-thio]triphosphate (GTPγS) (0.2-0.3 nM, 1250 Ci/mmol, Perkin Elmer) was added for an additional 30 min. The reaction was stopped after the transfer to a Millipore GF/C HTS^®^ microplate (ref. MSFCN6B50) by filtration of the incubation mix followed by three 250-µL washes. The filter-bound radioactivity was measured in a Microbeta TRILUX^®^ scintillation counter after 50 µL of scintillation liquid had been added (OptiPhase SuperMix, PerkinElmer). The binding of [^35^S]-GTPγS was determined for the reference agonist (R)-α-methylhistamine (maximal stimulation over basal set as 100%), and for the test compounds in the agonist mode, to calculate their half-maximal effective concentrations (EC_50_). Activity below basal, *i.e*., negative percentages, indicated inverse agonism. In the antagonist mode, the compounds were solubilized as stock solution in dimethyl sulfoxide (10^−2 ^M) and tested against (R)-α-methylhistamine-induced binding at its 80% maximal effective concentration (EC_80_) (obtained at 1 µM), to calculate their half-maximal inhibitory concentrations (IC_50_). EC_50_ and IC_50_ were determined with GraphPad Prism software version 9.0 (GraphPad Software LLC, San Diego, USA). The IC_50_ was used to determine functional affinity of each compound by calculating the Kb (Kb = IC_50_/(1 + ([Histamine]/EC_50_ of Histamine))) according to Cheng and Prusoff (1973).

### cAMP in whole cells

cAMP was measured in whole CHO-DUKX-MRE/CRE-Luc cells that express the human or mouse H3R (Acc #: NM_007232, NM_133849, respectively) using the HitHunter cAMP XS+ Assay (Discoverex, ref. 90-0075SM2) according to the manufacturer recommendations. Briefly, cells were grown in alpha-MEM complete medium (Gibco, ref. 12561-056) at +37 °C in a 95:5 air:CO_2_ atmosphere, collected after trypsin treatment (500 µg/mL trypsin + 200 µg/mL EDTA, Lonza, ref. BE17-161E), then centrifuged at 300 g for 15 min at +4 °C and resuspended in serum-free alpha-MEM medium containing 500 µM IBMX (ThermoFisher, ref. PHZ1124). 20,000 cells were distributed per well (Costar plate, half well, ref. 3882) in a 12.5-µL volume. In the agonist mode, 5 µL histamine was added in increasing concentrations for 15 min in the presence of IBMX 500 µM. Then 0.2 µM forskolin was added for an additional 30 min at +37 °C to calculate histamine EC_50_. In the antagonist mode, tested H3 antagonist compounds in increasing concentrations were pre-incubated for 15 min in the presence of IBMX 500 µM. Then, 0.2 µM forskolin was added with 100 nM histamine (corresponding to the mean histamine EC_80_) for an additional 30 min at +37 °C. 7.5 µL antibody reagent and 30 µL ED/Lysis/CL working solution of the HitHunter assay kit were added according to manufacturer recommendations and plates are incubated 1 h in the dark. Finally, 30 µL of cAMP solution A of the kit ware added prior incubation 3 h at room temperature. Luminescence was measured by the FDSS/μCELL apparatus (Hamamatsu Photonic). EC_50_ (for inverse agonism) and IC_50_ (for antagonism) were determined with GraphPad Prism. The IC_50_ was used to determine functional affinity of each compound by calculating the Kb (Kb = IC_50_/(1 + ([Histamine]/EC_50_ of Histamine))) according to Cheng and Prusoff (1973).

### H3R gene reporter assay

CHO-DUKX-MRE/CRE-Luc cells that expressed the human, mouse, or rat H3R (Acc #: NM_007232, NM_133849, NM_053506, respectively) were seeded overnight as 25,000 cells per well in a 96-well plate with white wall and clear flat bottom (Greiner, ref. 655098). Cells were washed twice with alpha-MEM serum-free medium (Gibco, ref. 12561-056) and wells were filled with 80 μL of medium. Serial 10X dilutions of compounds as well as forskolin 3 μM (10X concentrated) (Sigma, ref. F6686) were added, and cells were incubated at +37 °C in a CO_2_ incubator. Compounds and forskolin stocks were dissolved in DMSO, and final concentration of DMSO was 0.2%. In the antagonist mode, the compounds were tested against 100 nM of histamine. Following 4 h of incubation, the plates were taken out, the medium removed and replaced with 50 μL of luciferase reporter gene assay kit reagent (25 μL of Steady-Glo Promega, ref. E2510 + 25 μL of alpha-MEM). The microplate was gently shaken, and reagents allowed to react at room temperature for at least 10 min. Bioluminescence was measured using the FDSS/μCELL luminometer. Results were expressed as arbitrary forskolin-stimulated bioluminescence units. Data were analysed with GraphPad Prism to determine EC_50_ and intrinsic activity (i.a.) compared to histamine at full activity. The IC_50_ was used to determine functional Kb (Kb = IC_50_/(1 + ([Histamine]/EC_50_ of Histamine))) according to Cheng and Prusoff (1973).

### H3R calcium mobilization assay

HEK293 cells stably expressing the human H3 receptor (Acc# NM_007232) or CHO-DUKX-MRE/CRE-Luc expressing the mouse H3 receptor (Acc# NM_133849) were grown and maintained in DMEM-F12 (Lonza, ref. BE12-719F) or alpha-MEM media for HEK and CHO cells, respectively. Media were supplemented by 10% foetal bovine serum (Lonza, ref. DE14-801F), 100 U/mL penicillin/ 100 µg/mL streptomycin (Lonza, ref. DE17-602E), 1% glutamine (Lonza, ref. BE17-605E, 2 mM final concentration), 20 mM HEPES, pH 7.4 (Lonza, ref. BE17-737E) and 200 µg/mL of G418 sulphate (InvivoGen, ref. ant-gn-1) in 5% CO_2_ incubator at +37 °C. Cells were grown in 150-mm diameter plates. One plate (150 mm diameter) with adherent cells at sub-confluency was used per experiment performed in a 96-well plate (black plate, clear bottom, Costar, ref. 3904). The medium was removed 5 h prior to experiment and replaced by fresh medium. Cells are washed once by HBSS – Ca – MgCl_2_ buffer (Invitrogen, ref. 14185-045) supplemented by 20 mM HEPES buffer pH 7.4 (Lonza, ref. BE17-737E). Cells were detached by addition of trypsin solution (500 µg/mL trypsin and 200 µg/mL EDTA, Lonza, ref. BE17-161E). Equal volume of culture medium was added to inactivate trypsin, and cells were homogenized into single cell suspension. After centrifugation at room temperature, 200 x g 5 min, the cells were resuspended, washed once by 20 mL HBSS/HEPES buffer, and centrifuged again for 5 min at 200 x g. The cell pellet was resuspended in 16 mL of HBSS/HEPES buffer per 150 mm culture plate including 36 µL of working Fluo-4 solution: 50 µg Fluo-4-AM (Invitrogen, ref. F14201) were dissolved in 40 µL DMSO (Sigma, ref. D2650) and 32 µL of a 20% pluronic F-127 (solution in DMSO at 0.04% final concentration, Sigma, ref. P2443) were added to generate 72 µL of Fluo-4 working solution (1 mM Fluo-4 and 0.04% pluronic F-127 final concentrations). The buffer was supplemented by 16 µL of a 250 µM of water-soluble probenecid solution (Invitrogen, ref. P36400), 2.5 µM final concentration. The cell suspension was incubated for 40 min at +37 °C in a CO_2_ incubator and gently stirred every 10 min. Cells were then washed twice by HBSS/HEPES buffer to remove excess dye and resuspended in an appropriate volume of HBSS/HEPES buffer. Cells were counted and diluted in the same buffer to the desired density: 100 000 cells/160 µL for HEK-293 cells and 150 000/160 µL for CHO cells. In the agonist mode, 160 µL of the cell suspension in the 96-well plate. Additional incubation was performed in dark condition for 30 min at room temperature. Fluorescence was measured using the FDSS/μCELL. For agonism evaluation, 40 µL of 5X agonist compound in increasing concentrations were added per well containing 160 µL of cell (100 000 cells). In the antagonist mode, 20 µL of 10X H3R antagonists in increasing concentrations were added per well containing 140 µL of cell (100 000 cells) containing HBSS/HEPES buffer and were pre-incubated for 15 min at room temperature. Then, 40 µL of 5X histamine solution at EC_80_ concentration (100 nM) were injected into the 160 µL cell suspension per well. Responses were calculated by subtraction of the minimum fluorescence counts from the maximum fluorescence counts (F_max_ – F_min_). Results were normalized and were given as percentages of maximal response of the given histamine reference agonist. Data were analysed with GraphPad Prism to determine EC_50_ and intrinsic activity (i.a.) compared to histamine at full activity. The IC_50_ was used to determine functional Kb (Kb = IC_50_/(1 + ([Histamine]/EC_50_ of Histamine))) according to Cheng and Prusoff (1973).

### H3R beta-Arrestin2 assay

PathHunter^®^ CHO-K1 HRH3 β-Arrestin cell Line (DiscoverX, ref. 93-0509C2) overexpressing PK-tagged hH3R and EA-tagged β-Arrestin 2 was maintained in assay complete medium 107 from cell culture kit 107 (DiscoverX, ref. 92-3107 G) containing penicillin/streptomycin (100 U/mL penicillin and 100 µg/mL streptomycin, Lonza, ref. DE17-602E), hygromycin B (300 µg/mL) and geneticin (800 µg/mL) in 5% CO_2_ incubator at +37 °C. Cells were detached by addition of trypsin solution (500 µg/mL trypsin and 200 µg/mL EDTA, Lonza BE17-161E). Equal volume of culture medium was added to inactivate trypsin, and cells were homogenized into single cell suspension. After centrifugation at room temperature, 200 x g 5 min (Thermo/Jouan centrifuge, GR412), the cells were resuspended, washed once by 20 mL HBSS/HEPES buffer, and centrifuged again for 5 min at 200 x g The cell pellet was resuspended in AssayComplete™ Cell Plating 2 Reagent (DiscoverX, ref. 93-0563R2A).

For the assay, 20 000 cells were plated in 80 µL of Cell Plating 2 Reagent (PathHunter) into white µClear^®^ Cellstar^®^ culture 96-well microplate (Greiner bio-one, ref. 655098) and incubated for 16 h in 5% CO_2_ incubator at +37 °C. Compounds to be tested were diluted (stock solutions at 10^−2^ M) in HBSS 1X buffer (Gibco-Life Technologies, ref. 14065-049).

In the agonist mode, 20 μL of each 5X compound serial dilutions were added to the cells and the plate was incubated 90 min in 5% CO_2_ incubator at +37 °C. In the antagonist mode, 10 µL of each 10X antagonist serial dilutions, then 10 µL of 10 µM histamine solution (EC_80_) were added and the plate was incubated 90 min in 5% CO_2_ incubator at +37 °C. 50 µL of Working Detection Solution (19 parts of Cell Assay Buffer, five parts of Substrate Reagent 1 and one-part Substrate Reagent 2, DiscoverX PathHunter detection kit 93-001) were added followed by a 1-hour incubation at room temperature in the dark for the reaction to occur. The chemiluminescent signal was read on the 1450 microbeta TRILUX (PerkinElmer).

EC_50_s and IC_50_s for the antagonists were determined with GraphPad Prism. The IC_50_ was used to determine functional affinity of each compound by calculating the Kb (Kb = IC_50_/(1 + ([Histamine]/EC_50_ of Histamine))) according to Cheng and Prusoff (1973).

### Animals

Animals were housed in groups under a 12:12 h light/dark cycle (lights on at 7:00 a.m.) at a controlled temperature of +21 ± 2 °C and humidity of 45 ± 15% with free access to food (standard diet) and water, except when noted below. Experiments were conducted in accordance with European ethical standards (2013/118/EEC) and approved by local ethical committees (CEA n° 79 or CREMEAP). Male Wistar rats, male Long Evans rats and male Swiss mice were obtained from Janvier-Labs (Le Genest Saint-Isle, France). Male and female OF1 mice and male C57BL/6J mice were from Charles River (Saint-Germain-Nuelles, France). Female DBA/2J mice (age of 8–9 weeks at the beginning of experiments) were from Janvier Labs (Le Genest Saint-Isle, France). Mice were single housed for drinking in the dark test and ethanol self-administration experiments (cage of 370 cm^2^ and height 14 cm). For mouse behavioural experiments, all injections were made with a volume of 10 mL per kg of body weight by intraperitoneal (i.p.) route and sterile water was used for vehicle-treated mice.

### Effect on t-MeHA level in the brain of rodent

Male or female OF1 mice (20–25 g, Charles River) received received orally vehicle (methylcellulose 1%, 10 mL/kg), BP1.3656B (0.003, 0.01, 0.03, 0.1, 0.3 and 1 mg/kg), ciproxifan (1 or 3 mg/kg) or imetit alone (1, 3 or 10 mg/kg) or combined with BP1.3656B (0.02 mg/kg). Male Wistar rats (180–220 g, Janvier Labs) received received orally vehicle (methylcellulose 1%, 5 mL/kg) or BP1.3656B (0.03, 0.1, 0.3 and 1 mg/kg). Then, animals were sacrificed by decapitation at various times oral post dosing. The whole brain or the brain cortex were dissected out and homogenized in ten volumes (w/v) of ice-cold perchloric acid (0.4 N). The clear supernatant obtained after centrifugation (2000 x g, 30 min, +4 °C) was stored at −20 °C before measuring the t-MeHA level by enzymoimmunoassay as described [32].

### Ex vivo [^125^I]-iodoproxyfan binding to mouse brain membranes

Male OF1 mice (20–25 g) were fasted for 16 h before p.o. administration of BP1.3656A (0.03, 0.1, 0.3 or 1 mg/kg) between 9h00 and 10h00 a.m. Then, 90 min later mice were sacrificed, brains dissected out and homogenized in 5 mL of ice-cold buffer (Na_2_HPO_4_/KH_2_PO_4_ 50 mM pH 7.5). Binding experiments were performed according to Ligneau et al. (1994) with slight modification. Membrane preparations (50 µL membrane preparation/incubation) were incubated for 1 h at +37 °C in a final volume of 200 µL in the presence of 25 pM of [^125^I]-iodoproxyfan (Amersham 2 000 Ci/mmol). A 1 µM concentration of imetit, a specific H3-receptor agonist (Garbarg et al. 1992) allowed the determination of the non-specific binding. Incubations performed at least in triplicate were stopped by rapid filtration on Whatman GF/B glass fibre membranes. Radioactivity trapped on filters was directly counted using a gamma counter.

### Microdialysis

Male Wistar rats (275–325 g) were anaesthetized with chloral hydrate (Carlo Erba, 400 mg/kg, i.p.) and further mounted in a Kopf stereotaxic frame. A CMA/12 guide cannula (CMA Microdialysis; Phymep, Paris, France) was implanted into the hippocampus (AP, −5.5 mm from bregma; ML, +4.5 mm; DV, −4.0 mm from dura) according to the atlas of Paxinos and Watson [33] and further secured with dental cement and anchor screws into the skull. Animals were housed singly, and 5 days were allowed for postoperative recovery.

Between 9:00 and 10:30 a.m. on the day of the experiment, each animal was transferred into a freely moving animal system and a CMA/12 microdialysis probe (CMA Microdialysis; 3-mm membrane length) was slowly lowered into the guide cannula and allowed to recover for 2 h. Probes were perfused with artificial CSF perfusion fluid (CMA Microdialysis; 147 mM NaCl, 2.7 mM KCl, 1.2 mM CaCl_2_, 0.85 mM MgCl_2_, pH 7.4) at a flow rate of 1 µL/min (Harvard Apparatus, PHD 2000 syringe pump model). 50 nM of the acetylcholinesterase inhibitor, neostigmine, was added to the CSF perfusion fluid. The first three 30-minute dialysate samples were discarded, and the three subsequent ones were collected to provide a baseline. Ten minutes before the end of the basal fraction collection, vehicle (saline, 1 mL/kg), Pitolisant (10 mg/kg), BP1.3656B (0.03 and 0.1 mg/kg) were administered i.p. and six further 30-minute dialysates were collected.

### Electrochemical quantification of extracellular levels of acetylcholine (ACh)

The 30µL-dialysate samples were analyzed for ACh levels with HPLC-electrochemical detection (Waters, Saint-Quentin-en-Yvelines, France). The mobile phase (50 mM NaH_2_PO_4_, 500 µM EDTA and 0.5% ProClin in LC-grade water, pH 8.5) was pumped through an UniJet microbore ACh/Ch analytical column (530 × 1 mm, MF-8904, Bioanalytical Systems, Kenilworth, UK) coupled to an enzymatic post-column (50 × 1 mm) (MF-8903) at a flow rate of 0.13 mL/min. Eluates were quantified at +30 °C with the amperometric detector set at 500 mV (with a 3 mm platinum cell, an in situ Ag/AgCl reference electrode and a 50 µm spacer). A standard sample was included every 6-8 samples to enable quantification and check for reproducibility. The limit of detection for ACh was 3.0 fmol/µL.

### Effect on dopamine, noradrenaline, and serotonin turnovers in the mouse brain cortex

Male C57BL/6J mice (22–25 g, Charles River) received orally vehicle (methylcellulose 1%, 10 mL/kg), BP1.3656B (0.1, and 0.3 mg/kg). Ninety minutes later, they were killed by decapitation and brain cortex were dissected out, weighed, frozen in liquid nitrogen and stored at −80 °C. Tissues were homogenized in 4 mL of a 0.4 N perchloric acid / 2.7 mM ethylenediaminetetraacetic acid (EDTA) solution. After centrifugation (2000 x g, 30 min, +4 °C), supernatants were stored at −80 °C before analysis. Analysis were performed by use of high-pressure liquid chromatography (HPLC) coupled to electrochemical detection, according to the method of Ligneau et al. (2007a). Tissue concentrations of dopamine (DA), 3,4-dihydroxyphenyl acetic acid (DOPAC), homovanilic acid (HVA) serotonin (5-HT) and 5-hydroxyindole-3-acetic acid (5-HIAA), noradrenaline (NA) and 4-hydroxy-3-methoxy-phenylglycol (MHPG) were determined and the corresponding ratios (DOPAC/DA, HVA/DA, 5-HIAA/5-HT and MHPG/NA) were calculated.

### Pharmacokinetics in mice and rats

Male Swiss mice (23–25 g) received BP1.3656B (1 mg/kg, p.o. or 3 mg/kg, i.v. under a 10 mL/kg or 5 mL/kg administration volume, respectively) with methylcellulose (1%) or saline as vehicle. Male Wistar rats (200–220 g) received BP1.3656B (3 mg/kg, p.o. or i.v.) under a 5 mL/kg administration volume with methylcellulose (1%) in water or saline as vehicle. Plasma and brain concentrations of BP1.3656 were determined with liquid chromatography with tandem mass spectrometry analytical method. A generic internal standard was added to plasma and brain homogenates, and samples were extracted by use of Oasis^®^ HLB (Waters) solid-phase extraction plates. Eluted samples were separated on an Acquity^®^ UPLC BEHC18 column (Waters) at +50 °C using water/acetonitrile as the mobile phase (run time 3.5 min). The mass spectrometry ion source electrospray ionization was used in positive multiple reaction monitoring mode. The lower limit of quantification was 1 ng/mL (plasma) or 1 ng/g (brain) for the compound.

### Pharmacokinetic interactions between BP1.3656B and alcohol in mice and rats

Female DBA/2J mice (19–21 g) or male Long Evans rats (200–250 g) received BP1.3656B (0.3 mg/kg, i.p.) under a 10 mL/kg or 5 mL/kg administration volume, respectively with saline as vehicle. Thirty minutes later, animals received intraperitoneally alcohol 1.6 g/kg or 0.8 g/kg with saline as control under the same administration volume, respectively. Plasma concentrations of BP1.3656 were determined as described above and plasma alcohol concentrations were determined using an enzymatic assay (ref. n° 026, BioSentec, Portet sur Garonne, France).

### Behavioural studies

#### Alcohol self-administration in rats

Two cohorts with a total of 31 Long Evans male rats were used in this study. Fifteen were assigned to the Non-dependent group and were treated in a within subject design (n = 15 in each group). The remaining 16 rats were assigned to the Dependent group and were treated in a between subject design (n = 8 in each group). Regarding the sample size justification, for the non-dependent cohort, we used a within-subject design in which each animal received both placebo and BP1.3656B treatments (n = 15). This approach minimizes inter-individual variability and increases statistical sensitivity to detect moderate behavioral effects. In contrast, for the dependent cohort, a between-subject design was employed (n = 8 per group) because testing was not feasible after induction of dependence and relapse procedures. Based on pilot data and previous studies, we anticipated a markedly stronger effect of BP1.3656B in dependent animals compared to non-dependent ones. Indeed, our data confirmed a large effect size during relapse (Cohen’s d = 5.68 in Dep *versus* d = 1.30 in Non-Dep). Therefore, a smaller sample size in the Dep group was deemed sufficient to achieve adequate statistical power while adhering to the ethical principle of reduction (3Rs). A priori power estimations indicated that, for an expected large effect size (d  ≥ 1.5), a sample size of 7–8 animals per group would provide > 80% power (α = 0.05).

Rats were singly housed in individually ventilated cages with no enrichment in a 12 h/12 h light/dark cycle with the lights on at 7:00 a.m., food and water available ad libitum and temperature controlled at +21 ± 2 °C. A double-blind design was followed, and drugs (BP1.3656B and placebo) were put in solution in 0.9% saline 5 to 10 min prior to the intraperitoneal injections and administered following a Latin square design (except experiments in non-dependent rats), thus each rat was its own control.

#### Acquisition of the operant task

All the rats (15 non-dependent and 15 dependent) were submitted to the same procedure to acquire the operant task. After 8 weeks of a 20% ethanol intermittent access protocol (2-bottle choice paradigm with water *versus* a 20% ethanol solution) rats were submitted to a schedule of training (Jeanblanc et al., 2018) to finish with 15 min sessions during which rats need to press 3 times to obtain the reward, namely a 0.1 mL drop of a 20% ethanol (v/v) solution. After the rats reached a stable baseline of consumption the non-dependent rats directly enter the procedures of injection of the compounds while the dependent rats were submitted to the procedure of inhalation of alcohol vapours.

#### Inhalation of alcohol vapours

The 15 rats assigned to the dependent group were submitted to inhalation of alcohol vapours (40 mg/L of air) 12 h per day for 10 weeks to induce dependence. Rats continued their training of self-administration in the operant cages twice a week (Tuesdays and Fridays) during the inhalation period but 6 h after the end of the daily exposure to vapours).

#### Effects of drugs on motivation, extinction, and relapse

To evaluate the effect of the compounds on the motivation to consume alcohol rats were submitted to a progressive ratio protocol in which the effort needed to obtain a single dose of alcohol increases after each delivery of alcohol (3 presses to obtain the 1^st^ reward, 4 presses for the 2^nd^, 5 for the 3^rd^ and so long following this increment: 3, 4, 5, 7, 9, 12, 15, 17, 20, 22, 25, 28, 30, 33, 35). In the extinction procedure, BP1.3656B and its placebo were injected 1 h prior to the beginning of the dark phase of the day (6:00 p.m.) and the self-administration sessions were performed the following day at 9:00 a.m. During these consecutive daily sessions of extinction of the operant self-administration behaviour, the presses on the active lever led to no events (no light cue and no delivery of alcohol). We did not observe any increase in the number of inactive lever presses during these sessions. We considered that rats extinguished their behaviour when they pressed less than 20% of the baseline level of active lever presses. After 6 extinction sessions, BP1.3656B and its placebo were injected 30 min prior to the self-administration session to test their effect on relapse. The relapse was tested using a re-acquisition protocol in which the presses on the active lever (fixed ratio 3) led again to the occurrence of the light cue and to the delivery of alcohol. To initiate the relapse a “free” drop of ethanol was provided at the beginning of the 1^st^ re-acquisition session.

#### Ethanol-induced locomotor activity in mice

Locomotor activity of female DBA/2J mice was recorded in individual black plastic open fields (40 × 40 × 30 cm height) for single administration experiments, or transparent plastic open fields (26 × 42 × 20 cm height) for repeated administration experiments. Open fields were in a dimly lit room (100 lux) with a continuous masking noise (70 dB) and a video-tracking system (Ethovision XT4.1/10.1 Noldus, Wageningen, the Netherlands) allowed behavioural analyses based on centre-point detection. Single administration experiments were performed between 9:00 a.m. and 5:00 p.m. Mice were placed individually in the open fields for a 90-minute habituation period. Then, animals received a pre-treatment with vehicle or BP1.3656B (0.005, 0.03 or 0.3 mg/kg) and 30 min later saline or ethanol (1.6 g/kg). Locomotor activity analysis was performed for 5 min after ethanol injection providing the distance moved. Repeated administrations experiments were performed between 9:00 a.m. and 1:00 p.m. Mice received a saline injection immediately prior placing them in the open fields for a 5-minute habituation session. Then, mice were assigned to saline, or ethanol group counterbalanced for baseline locomotor activity determination. During four consecutive days, mice were given one daily injection of saline, or ethanol (1.6 g/kg) immediately followed by a 5-minute locomotor activity session in the open fields. Vehicle or BP1.3656B (0.3 mg/kg) were administered 30 min before saline or ethanol injection. Effects of BP1.3656B on acquisition of ethanol-induced behavioural sensitization, was assessed in animals receiving vehicle or BP1.3656B from day 1 to day 4.

#### Drinking in the dark in mice

The behavioural drinking in the dark (DID) paradigm described by Rhodes et al. (2005) was used with the following modifications. Two weeks before DID experiments, light / dark cycle was reversed (lights on 11:00 p.m.). During this period, male C57BL/6J mice were trained to drink water in a two-bottle free choice procedure using two plastic bottles all affixed with ball-bearing sipper. Then, for ethanol habituation, one bottle was successively filled with ethanol 5% and 10% (v / v) for 3 days and 2 days, respectively to avoid ethanol aversion. After ethanol habituation, the ethanol bottle was replaced by a water bottle and ethanol 10% access was only allowed for daily 2-hour binge drinking sessions 3 h after lights off in a two-bottle free choice procedure. One week was required to achieve a stable ethanol drinking consumption. Then, mice were given access to the 2-hour two-bottle choice binge drinking sessions four times a week on days 1 to 4. Pre-treatments were administered only on alternate days (days 1 and 3 without injection, days 2 and 4 with injection) to avoid any association which could develop when always pairing ethanol solution with injection. Vehicle or BP1.3656B (0.3 mg/kg) were given 30 min before access to ethanol solution. After each binge drinking session, fluid intakes (volume in mL) were measured, and ethanol bottles were replaced by water bottles. For the drinking in the dark paradigm using sucrose 10%, a similar protocol was used; except that the sucrose habituation was performed directly with sucrose 10% as no aversion occurred.

#### Ethanol self-administration in mice

Male C57BL/6J mice were given free access to ethanol in a two-bottle free choice paradigm for 24 to 29 days where one water bottle and one ethanol bottle were available ad libitum. Ethanol concentration was fixed at 5% during the first week and then raised to 10%. Control mice had access to two bottles filled with water. Fluid consumption was recorded every 3 or 4 days and positions of the ethanol and water bottles were alternated simultaneously to prevent side preference bias. Mice were weighed once a week during the alcohol consumption period and habituated to i.p. injections the last week. After the free-choice period ethanol drinking, ethanol 10% bottles were removed for ethanol withdrawal (ethanol-WD) groups before evaluation of withdrawal symptoms in behavioural test. A no-withdrawal (no-WD) group was still allowed to self-administer ethanol in the two-bottle free-choice procedure until behavioural test. Control mice were provided only water throughout the study duration.

#### Elevated plus maze test in mice

The elevated plus maze (EPM) test, typically used to measure anxiety-like behaviour (Belzung and Griebel, 2001), was chosen to evaluate anxiety 24 h after ethanol withdrawal. It was performed between 1:00 p.m. and 5:00 p.m. Mice were tested on a white EPM in a dimly lit room (100 lux) with a continuous masking noise (70 dB). EPM apparatus consisted of two open arms (35 × 5 cm) and two closed arms (35 × 5 × 15 cm height) arranged perpendicularly and elevated 50 cm above the floor. Test sessions lasting 5 min started with a mouse introduced in the central platform of the EPM facing a closed arm.

Vehicle or BP1.3656B (0.3 mg/kg) were given 20 h and 30 min before EPM test. Times spent in the open arms and closed arms of the maze as well as the distance moved in each arm were determined with the video-tracking system.

#### Statistics for preclinical studies

Statistics were calculated with GraphPad Prism 7.0 (GraphPad Software, San Diego, California USA). The statistical significance of differences between experimental groups was assessed by paired Student-Fisher t-tests or analysis of variance (ANOVA), and *p* < 0.05 was taken as the threshold of significance.

#### Drugs

BP1.3656B ((S)-4-(4-(3-(3-methylpiperidin-1-yl)propoxy)phenyl)pyridine 1-oxide, dihydrochloride salt, tetrahydrate), BP1.3656A (as an oxalate salt) were synthesized by the Bioprojet-Biotech Medicinal Chemistry Department and its synthesis will be described in another article. Pitolisant (BF2.649 hydrochloride salt), ciproxifan, imetit and (R)α-methylhistamine were obtained from Bioprojet (Paris, France). Histamine was obtained from Sigma-Aldrich (Saint-Quentin Fallavier, France). Absolute ethanol was obtained from VWR Chemicals (Fontenay-sous-Bois, France) and its density was considered for doses calculations. Absolute ethanol was obtained from VWR Chemicals (Fontenay-sous-Bois, France) and its density was considered for doses calculations. Except when indicated, drug doses were expressed as free bases.

#### Clinical studies

Informed consent was obtained from all study subjects in each of the clinical studies.

#### Phase I clinical studies in healthy subjects: acute and subchronic administration

These studies were the first administration of BP1.3656B in human participants.

For acute administration, the first study was single centre, randomized, double-blind, placebo-controlled, single oral dose, with ascending doses of BP1.3656B from 1 to 150 µg. This study was approved by the Comité de Protection des Personnes (CPP) Sud Est III (Ethics Committee) Lyon (France). Since no data was available for the calculation of the sample size, it was decided to include 48 subjects (12 per part; 8 active, 4 placebo), which is the usual number of subjects for such studies. Forty-five healthy young adults were included (48 observations with 3 subjects participating to two different study parts) with 12 subjects per dose (8 subjects receiving active treatment and 4 receiving placebo). Each subject being receive after randomisation, three treatments (two single dose of BP1.3656B or placebo). The objectives of this first study, were to assess the pharmacokinetics, the safety and the pharmacodynamic profile (sleep diary) of BP1.3656B.

For chronic administration, the second study was a double-blind, placebo controlled, 10-day multiple ascending dose study. This study was approved by the Comité de Protection des Personnes (CPP) Sud-Est IV (Ethics Committee) Lyon (France). Since no data was available for the calculation of the sample size, it was decided to include nine subjects per dose (6 verum, 3 placebo), which is the usual number of subjects for such studies. BP1.3656B was administered once a day, at two dose levels (60 μg and 90 μg) for 10 days, in 12 healthy volunteers to determine PK and safety profiles of BP1.3656B and to explore possible pharmacodynamic (PD) effect of BP1.3656B on sleep and wake by polysomnography (PSG) and electroencephalogram (EEG).

For chronic administration, the third study was a double-blind, placebo controlled, 21-day multiple ascending dose study. This study was approved by the independent Ethics Committee (CPP, Comité de Protection des Personnes) of Sud-Est IV (Lyon - France). The study followed a single ascending dose schedule with a maximum of 3 dose levels. For each dose level a cohort of 12 subjects were randomized, 9 received active substance and 3 received placebo. An additional group of 12 female subjects was randomized after determination of MAD or MTD dose, 9 received active substance and 3 received placebo. This sample size was deemed acceptable to allow a good evaluation of the safety and its changes across dose levels and compared to placebo. Evaluation of the impact of BP1.3656B administration on QTc prolongation was investigated through modelling of the relationship between BP1.3656 (or active metabolite, once identified) serum concentration and contemporary QTc measurements. Based on the study design a set of 1152 individual pairs of “serum concentration; QTcF value in triplicate” for each compound were then available (including placebo data). Moreover, these sets of data should cover an extended range of serum concentrations (low concentrations in the late post-dose PK points and high concentrations around Cmax). Therefore, the sample size appeared appropriate for this analysis. Same justification was also applicable for moxifloxacin. To validate the methodology and calibrate the study for QTcF assessment, each subject received a 400 mg dose of moxifloxacin under open-label conditions on Day −8. This was followed by a BP1.3656B placebo administration on Day 1, and subsequently by a 21-day period of repeated administration of either BP1.3656B or placebo, from Day 1 to Day 21, under blinded conditions. Thirty-six male subjects were studied in 3 groups of 12 subjects. In each group, 9 subjects received BP1.3656B for 21 days and the 3 remaining subjects received placebo for 21 days. Three dose levels of BP1.3656B (30 µg, 60 µg and 90 µg) were administered. A fourth group of 12 female subjects received a 60 µg dose (9 receiving BP1.3656B and 3 placebo).

#### PET study

This PET study was designed to determine the occupancy of the H3R by BP1.3656B after acute and chronic administration. This study was approved by the Research Ethics Board at the Centre for Addiction and Mental Health – CAMH (REB#103/2014). The sample size was estimated by considering the findings of previous studies and sample sizes used therein. In two published reports using [^11^C]-GSK189254 to measure occupancy of H3 receptors [34, 35], sample sizes were about 5–7 subjects. In both published reports, statistical significance was obtained with samples of this size the standard deviations reported in the two studies were comparable. Thus, the proposed sample size of 8 was chosen to safely observe any effects if they existed.

For acute administration: a within-subjects, single-blind, fixed-order drug schedule design was used to characterize the dose-response occupancy of BP1.3656 at the H3R. In 4 participants, BP1.3656B or placebo was administered orally at 0 µg (placebo) then 30 µg on separate days. In random order, participants then received either 60 µg or were scanned 24 h after an acute dose of 30 µg. Injection of the PET tracer [^11^C]-GSK189254 was timed 180 min after dosing with food and water. For scans that occurred 24 h after the last dosing, participants were given food and water 3 h prior to the scan. Blood samples were taken at dosing, 180 min and 270 min after dosing. For the scan that occurred 24 h after the last dose of BP1.3656B, blood samples were taken 3 h prior to the injection, at the time of the injection and at the end of the scan.

For subchronic administration: For the sub chronic dosing study, participants were given BP1.3656B (30 µg or 60 µg) sub chronically for 5 to 10 days and then scanned with [^11^C]-GSK189254 either 3 h or 24 h after dosing. For the 30 µg experiment, participants were first scanned following placebo administration and then took 30 µg BP1.3656B for 5 to 10 days. Four participants were scanned 3 h after the last dose and a different 4 participants were scanned 24 h after the last dose. For the 60 µg study, 4 participants were first scanned following placebo then scanned 3 h after the last dose of sub chronically administered BP1.3656B (60 µg). The third scan was 24 h after the last dose of 60 µg of BP1.3656B.

The total volume of distribution (V_T_) were estimated with a 2 tissue compartment model using arterial input function. K1/k2 were coupled across regions during fitting of the kinetic model as we have done previously [22]. V_T_ for brain regions of interest are presented as mean ± s.e.m. As usual for radioligands quantified using V_T_, individual occupancy was determined using the Lassen Plot [36, 37]. Lassen plot assumes that the V_T_ of the non-displaceable compartment (free and non-specific binding) and the occupancy is the same for all the region of interest analyzed. PET scanning with [^11^C]-GSK189254 is susceptible to mass dose effects. Methodological considerations that can limit the mass dose effect are injecting the lowest possible mass of the radioligand, with the highest specific activity possible. In the present study this was done, and application of the Gallezot et al. correction [38] did not yield appreciably different results from the apparent occupancies provided. In addition, percent apparent occupancy is provided below (Fig. 3e) for individual participants, with mean and s.e.m.

#### Intravenous self-administration studies in subjects with AUD

This study was approved by the Research Ethics Board at the Centre for Addiction and Mental Health – CAMH (REB#072/2017) and was registered on ClinicalTrials.gov under identifier NCT04727086. Therapeutic potential of BP1.3656B was evaluated by examining medication effects on alcohol motivation and self-administration in a placebo-controlled human laboratory study with IV-ASA. IV-ASA affords exceptional experimental control and is a promising method for AUD medication screening studies [39]. Because no human data on BP1.3656 was available to estimate medication effect sizes, a power analysis was conducted assuming a small-to-medium medication effect size (f = 0 .20). A within-subjects design with a within-subjects correlation of 0.7 across medication and placebo sessions were selected. With these assumptions and an alpha value of 0.05, a final sample of N = 32 was chosen to afford good power (0.80) to detect a significant medication effect on the IV-ASA outcomes. Non-treatment-seeking adults who met criteria for past-year AUD were recruited from the community (492 screened, 36 randomized; see Table S04 for participant characteristics). Following eligibility screening and clinical assessment participants were randomized to treatment order (BP1.3656 first or placebo first) prior to sub-acute treatment with BP1.3656B and placebo in a double-blind, within-subjects, counterbalanced design. Each treatment phase had a target duration of at least 10 days (medication: M = 13.07 (SD = 1.33) days, range: 10–14; placebo: M = 12.81 (SD = 2.01) days, range: 9–14), separated by a 14-day washout period. Each treatment phase concluded with two laboratory visits involving operant IV-ASA procedures (up to four IV self-administration sessions total), consisting of one free-access (FA) IV-ASA session and one progressive ratio (PR) IV-ASA session phase (fixed order with sessions separated by at least one day). FA and PR sessions served as laboratory assays of alcohol liking and alcohol motivation/wanting, respectively [40]. IV self-administration procedures were carried out in a research hospital setting using the computer-assisted alcohol infusion system (CAIS), as described previously [39, 41]. During FA sessions (120 min) participants voluntarily self-administered (6% ethanol in saline) at their discretion using button presses on a fixed-ratio (FR1) schedule. Each alcohol request triggered a 2.5-min infusion at a rate (calculated by CAIS in real-time using pharmacokinetic modeling) to achieve an incremental arterial blood alcohol concentration (BAC) increase of (7.5 mg%) per request. An upper safety limit of 180 mg% was imposed. PR sessions (180 min) were similar to FA sessions, but involved operant self-administration using a progressive workset necessitating an escalating number of button presses to earn successive infusions. The PR schedule ensured an inevitable decline in BAC approximately midway through the session, providing a context to measure effort expenditure as alcohol availability and BAC declined [42]. Both FA and PR sessions began with a standardized priming phase (target BAC: 30 mg%). Given the focus on within-person effects of medication, sessions did not incorporate a non-alcohol (saline-only) control.

#### Randomized clinical trial testing BP1.3656B to reduce alcohol consumption in subjects with AUD

This study was registered on ClinicalTrials.gov under identifier NCT03424824. This study was approved by the Ethics Committee for multicenter trials of Sofia (Bulgaria), by the Comité de Protection des Personnes (CPP) Nord-Ouest I of Rouen (France) and by the Ethics Council at Ministry of Healthcare of the Russian Federation of Moscow (Russia). This was a Group Sequential Test (GST) multicenter, randomized, double-blind, placebo-controlled phase II trial with parallel groups to evaluate the effectiveness and the safety of BP1.3656B 30 μg or 60 μg or 90 μg OD compared to placebo in reducing alcohol consumption during a 12-week double blind treatment followed by a one-week wash out period under single blind placebo. Eighteen active centers within 3 countries (France, Bulgaria, Russia) participated in the trial.

The primary end point was the decrease in number of monthly heavy drinking days (HDD) (≥ 60 g/day in men and ≥ 40 g/day in women) from baseline to end of the double-blind Randomized Treatment (RT). Secondary endpoints were designed to assess safety and tolerability and to further investigate the effect of BP1.3656B on other alcohol use criteria (*e.g*., total alcohol consumption, number of abstinence days), craving (OCDS questionnaire) as well as the improvement in mental health (depression, sleep with SF-12 Health Survey and Pittsburgh Sleep Quality Index) and quality of life. Total Alcohol Consumption (TAC) was used as one of the secondary outcomes. Both HDDs and TAC were measured through the Time Line Follow Back (TLFB). Other secondary endpoints included: Continuous Controlled drinking (CCD, a binary endpoint defined as no HDDs in the 12 weeks medication period), Percent of Abstinent days (PAD, percentage of days without any drink in the 12-week medication period) and Continuous Abstinence Duration (CAD a binary endpoint defined as PAD = 100). Safety was assessed notably by evaluation of treatment emergent adverse events (TEAE), physical examinations and clinical laboratory tests.

Regarding sample size calculation, by considering a slightly higher SD = 8 and a baseline-final correlation of R = 0.6 – a significant benefit of at least 3.5 days compared with the placebo could be detected with a power of 80% at one tailed alpha risk of 5%, when at least 40 patients were available in each treatment group. The planned GST included 3 looks based on an expected difference of 3.5 days a one tailed test at 0.05 one-sided significance. The initial sample size for a fixed length study was 40*3 = 120 patients. Using three looks in maintaining the same conditions and controlling for type 1 error, a total of 129 patients were needed. The 3^rd^ look was performed in the GST at 124 patients and did not reveal conditions of early interruption for either success or futility. In accordance with the SAP of June 25th, 2020, a new dosage of 90 μg was introduced. By considering a SD of 8 and a baseline-final correlation of R = 0.6 (these values correspond to the blind analysis after the GST at n = 124 patients), a significant benefit of at least 3 days nHDD for the 90 μg compared with the placebo could be detected with a power of 80% at one tailed alpha risk of 5%, when at least 60 patients are available in each group. The sample size per group was ≅ 43 patients. Placebo group was added of 20 patients (n ≅ 63) and the new dosage was studied on 60 patients. The approximate sample size for each group was 63, 43, 43, 60, for placebo, 30 μg, 60 μg and 90 μg treatment groups respectively, thus a total of 209 patients were needed. Patients were male and female patients with moderate to severe AUD with self-report of at least 15 HDDs or more ( ≥ 60 g/day in men & ≥ 40 g/day in women) during the 30 days prior to screening and 7 HDDs or more during the 2 weeks between screening and baseline. The study was performed in accordance with the Ethical principles stated in the Declaration of Helsinki (current version), the international recommendation of Good Clinical Practices (ICH-E6) as well as with the local regulation. In addition to IRB approval, the study was also under an independent Data Safety Monitoring Board. If the experimental treatment does not provide at an early point any evidence of its potential beneficial effect in alcohol dependence, it would be unethical to continue the trial and better interrupt the useless exposure of patients. A Group Sequential Test was chosen allowing stopping the trial either: (1) when the observed difference already provides convincing evidence of a significant effect of the tested drug, (2) when the observed difference is so tiny that the conditional power at the end of the trial is too small. At each look, if conditions (1) or (2) are not found, a new look is decided, and so on until the last planned look. The 3^rd^ look was performed in the GST at 124 patients and did not reveal conditions of early interruption for either success or futility in accordance with the Statistical Analysis Plan of June 25^th^, 2020, a new dosage of 90 μg was be introduced.

The study was conducted in two parts:

1. Patients were 1^st^ randomised 1/1/1 during the 1^st^ part of the study (BP1.3656B 30 μg o.d. or 60 μg o.d. *versus* placebo, respectively).
2. In accordance with the Statistical Analysis Plan (SAP) of June 25^th^, 2020, the 90 μg dosage of BP1.3656B was introduced in the 2^nd^ part of the study and patients were randomised 3/1 (BP1.3656B 90 μg o.d. *versus* placebo, respectively).

The approximate sample size for each group was 63, 43, 43, 60, for placebo, 30 μg, 60 μg and 90 μg treatment groups respectively, thus a total of 209 patients were needed.

## Supplementary information

Extended Data

## Data Availability

The data that support the findings of this study are not publicly available but may be obtained from the corresponding author upon reasonable request.
