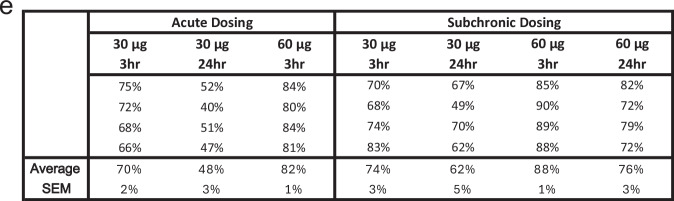# Correction: Histamine H3 Receptor as a target for alcohol use disorder: challenging the predictability of animal models for clinical translation in drug development

**DOI:** 10.1038/s41398-026-03931-9

**Published:** 2026-03-16

**Authors:** Bernard Le Foll, Mickael Naassila, Jérôme Jeanblanc, Christian S. Hendershot, Jesus Chavarria, Thierry Calmels, Stéphane Krief, Isabelle Berrebi-Bertrand, Marilyne Uguen, David Perrin, Xavier Ligneau, Isabelle Boileau, Pablo M. Rusjan, Patricia Di Ciano, Pamela Sabioni, Marc Capet, Philippe Robert, Olivier Finance, Jeanne-Marie Lecomte, Jean Charles Schwartz

**Affiliations:** 1 Translational Addiction Research Laboratory, Campbell Family Mental Health Research Institute, Centre for Addiction and Mental Health, University of Toronto, Toronto, ON Canada; 2 Institute for Mental Health Policy Research, Centre for Addiction and Mental Health, Toronto, ON Canada; 3 Department of Family and Community Medicine, University of Toronto, Toronto, ON Canada; 4 Department of Pharmacology and Toxicology, University of Toronto, Toronto, ON Canada; 5 Institute of Medical Sciences, University of Toronto, Toronto, ON Canada; 6 Department of Psychiatry, University of Toronto, Toronto, ON Canada; 7 Campbell Family Mental Health Research Institute, Toronto, ON Canada; 8 Université de Picardie Jules Verne, Groupe de Recherche sur l’Alcool & les Pharmacodépendances, INSERM UMR1247, Amiens, France; 9 Department of Population and Public Health Sciences and Institute for Addiction Science, Keck School of Medicine, University of Southern California, Los Angeles, CA USA; 10 Department of Psychology, Western University, London, ON Canada; 11 Bioprojet-Biotech, Saint-Grégoire, France; 12 Brain Health Imaging Centre, Centre for Addiction and Mental Health, Toronto, ON Canada; 13 The Douglas Research Centre, McGill University, Montreal, QC Canada; 14 Dalla Lana School of Public Health, University of Toronto, Toronto, ON Canada; 15 Bioprojet-Pharma, Paris, France

**Keywords:** Neuroscience, Drug discovery, Addiction, Psychiatric disorders, Health sciences

Correction to: *Translational Psychiatry* 10.1038/s41398-026-03807-y, published online 29 January 2026

The version of Figure 1 that was incorporated lacked several panels.

In addition, the table at the end of Figure 3 was incomplete, as several mean and standard error of the mean values were missing from the bottom. The original article has been corrected.

Incorrect Figure 1
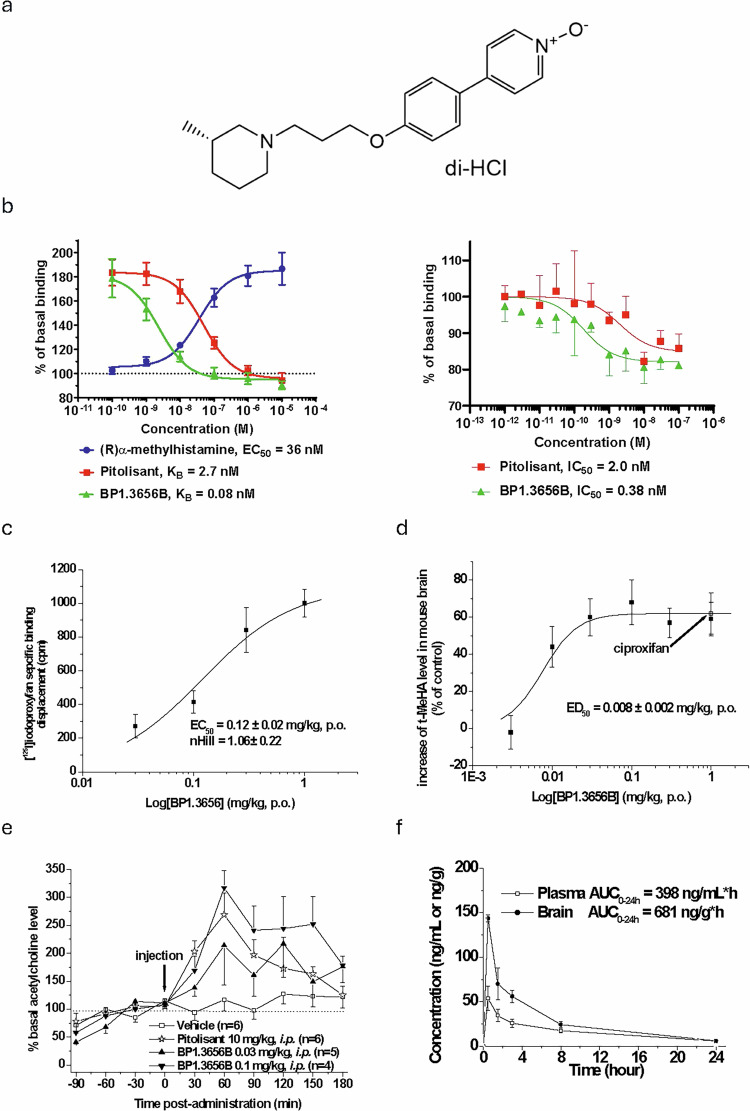


Correct Figure 1
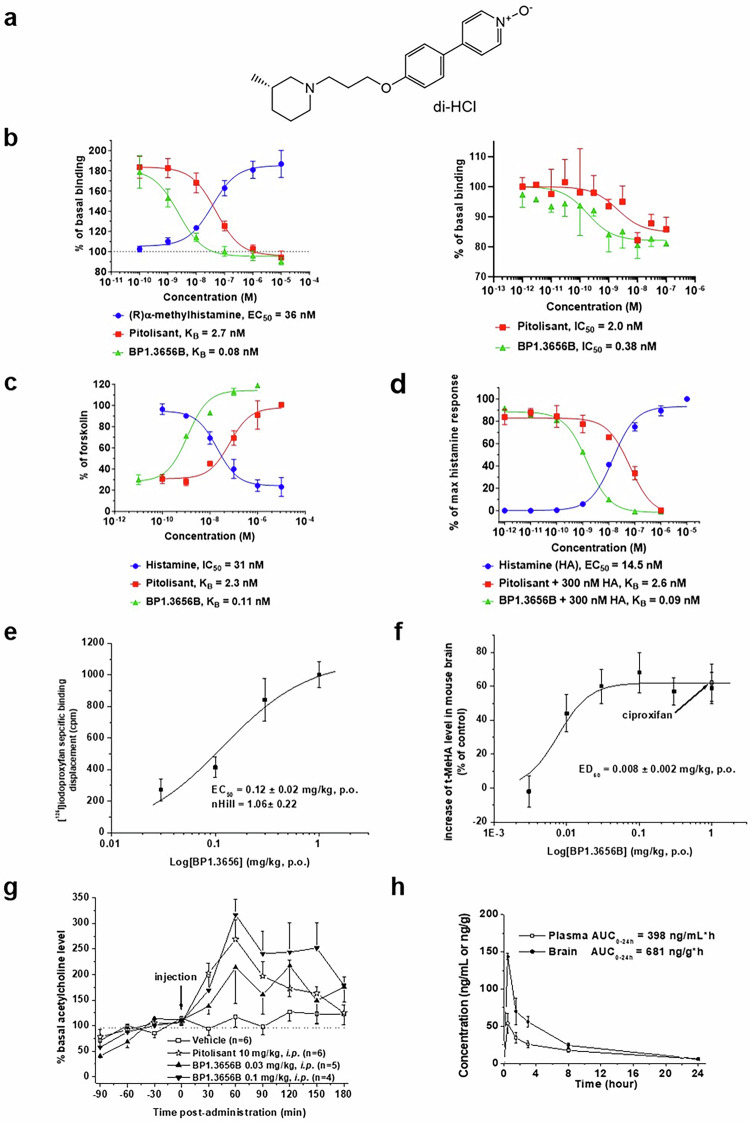


Incorrect Figure 3e
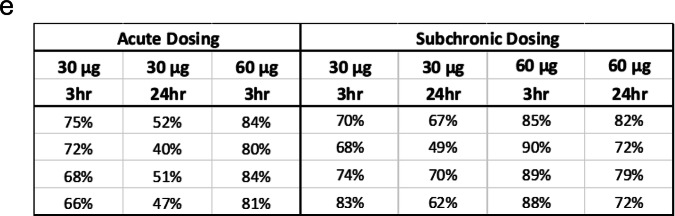


Correct Figure 3e