# Supplementary material for: Histamine H3 Receptor as a target for alcohol use disorder: challenging the predictability of animal models for clinical translation in drug development
Source: Transl Psychiatry. 2026 Jan 29;16:55. doi: 10.1038/s41398-026-03807-y (PMC12873132; doi:10.1038/s41398-026-03807-y)
Supplement: Supplementary file 1 — Extended Data [file 41398_2026_3807_MOESM1_ESM.docx]

**Extended Data**

Table S01. a) Affinity and efficacy of BP1.3656B at the human recombinant histamine H3 receptor; b) Affinity and efficacy of BP1.3656B at naïve and recombinant mouse and rat histamine H3 receptors.


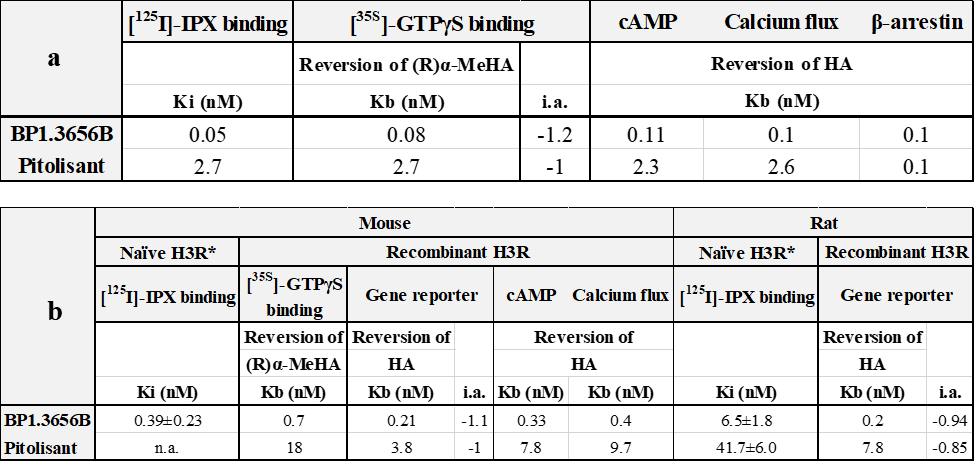


Table S02: Pharmacokinetics of BP1.3656 and alcohol in female DBA/2J mice receiving vehicle or BP1.3656B (0.3 mg/kg, i.p.) 30 minutes before alcohol (1.6 g/kg, i.p.) or saline.

|  | Plasma concentration | | | |
| --- | --- | --- | --- | --- |
|  |  | | | |
| Time post alcohol dosing | 10 minutes | | 30 minutes | |
|  | BP1.3656 (ng/mL) | Alcohol (g/L) | BP1.3656 (ng/mL) | Alcohol (g/L) |
| BP1.3656B + saline | 30.57 ± 1.42 | - | 22.23 ± 0.89 | - |
| BP1.3656B + alcohol | 25.76 ± 1.79 | 2.05 ± 0.09 | 24.62 ± 2.12 | 1.60 ± 0.12 |
| Vehicle + alcohol | - | 2.09 ± 0.03 | - | 1.59 ± 0.11 |

Mean ± SEM of 6 mice per condition.

Table S03: Summary of descriptive statistics for PK parameters of BP1.3656 by dose level. a. Study P08-04 (SAD), b. Study P10-02 (MAD for 10 days), c. Study P14-03 (MAD for 21 days)

| 1. Study#1 | | P08-04 (SAD) | |  |  | |  | |  | |  | |  | |  |
| --- | --- | --- | --- | --- | --- | --- | --- | --- | --- | --- | --- | --- | --- | --- | --- |
|  | |  |  |  |  | |  | |  | |  | |  | |  |
| **PK parameters** | | **Period 1** | **Period 2** | **Period 3** | | **Period 4** | | **Period 5** | | **Period 6** | | **Period 7** | |  |  |
| **Mean ± SD CV%** | | **1µg** | **3µg** | **10µg** | | **30µg** | | **60µg** | | **100µg** | | **150µg** | |  |  |
| **C_max_** | | **n.c.** | **n.c.** | **21.79 ± 5.45** | | **55.51 ± 18.01** | | **111.88 ± 30.44** | | **177.75 ± 17.66** | | **280.60±101.77** | |  |  |
| **(pg/mL)** | |  |  | **25.02** | | **32.44** | | **27.21** | | **9.93** | | **36.27** | |  |  |
| **T_max_** | | **n.c.** | **n.c.** | **3.94 ± 2.01** | | **2.81 ± 0.53** | | **2.50 ± 1.25** | | **4.13 ± 1.81** | | **3.50±2.12** | |  |  |
| **(h)** | |  |  | **50.99** | | **18.86** | | **50.14** | | **43.82** | | **60.61** | |  |  |
| **T_1/2_** | | **n.c.** | **n.c.** | **11.24 ± 0.61§** | | **14.50 ± 4.68** | | **15.54 ± 5.49** | | **16.41 ± 1.69** | | **18.55± 5.29** | |  |  |
| **(h)** | |  |  | **5.41** | | **32.23** | | **35.35** | | **10.27** | | **28.52** | |  |  |
| **AUC_0-48h_** | | **n.c.** | **n.c.** | **156.91 ± 36.73** | | **861.73 ± 468.84** | | **1766.91 ± 509.14** | | **3273.47 ± 250.39** | | **5338.96± 659.82** | |  |  |
| **(pg/mL.h)** | |  |  | **23.41** | | **54.41** | | **28.82** | | **7.65** | | **12.36** | |  |  |
| **AUC_0-∞_ (pg/mL.h)** | | **n.c.** | **n.c.** | **369.57 ± 169.36** | | **1212.02 ± 570.46** | | **2141.30 ± 571.74** | | **3807.96 ± 359.73** | | **6490.13±920.96** | |  |  |
|  | |  |  | **45.83** | | **47.07** | | **26.7** | | **9.45** | | **14.19** | |  |  |
|  | n.c.: not calculated (Main of BP1.3656 concentration values were below the limit of quantification) | | | | | | | | | |  | |  | |  |
|  | §: determined with data values from only two subjects. | | | | | |  | |  | |  | |  | |  |
|  | |  |  |  |  | |  | |  | |  | |  | |  |

| 1. Study#2 | P10-02 (MAD for 10 days) |  |  |  |  |  |  |
| --- | --- | --- | --- | --- | --- | --- | --- |
|  |  |  |  |  |  |  |  |
| **PK parameters** | **Cohort 1** | | | **Cohort 2** | | |  |
|  | **Dose level 60 µg** | | | **Dose level 90 µg** | | |  |
| **Mean ± SD** |  | | |  | | |  |
| **CV%** |  | | |  | | |  |
| **Day** | **1** | **10** | **Racc** | **1** | **10** | **Racc** |  |
| **C_max_** | **121.33 ± 30.45** | **172.44 ± 40.30** | **1.42** | **171.29 ± 31.34** | **344.32 ± 55.26** | **2.01** |  |
| **(pg/mL)** | **25.1** | **23.4** |  | **18.3** | **16** |  |  |
| **T_max_** | **2.50 ± 0.77** | **3.17 ± 1.57** | **n.a.** | **3.42 ± 1.36** | **2.75 ± 0.61** | **n.a.** |  |
| **(h)** | **31** | **49.6** |  | **39.7** | **22.3** |  |  |
| **T_1/2_** | **n.c.** | **32.2 ± 5.2** | **n.a.** | **n.c.** | **34.3 ± 3.9** | **n.a.** |  |
| **(h)** |  | **16** |  |  | **11.5** |  |  |
| **AUC_0-τ_** | **1365 ± 269** | **2356 ± 305** | **1.73** | **2368 ± 452** | **5049 ± 824** | **1.73** |  |
| **(pg/mL.h)** | **19.7** | **13** |  | **19.1** | **16.3** |  |  |
| **AUC_0-t_** | **n.a** | **4023 ± 679** | **n.c.** | **n.a** | **9238 ± 1497** | **n.c.** |  |
| **(pg/mL.h)** |  | **16.9** |  |  | **16.2** |  |  |
| **Clss/F** | **35333 ± 5087** | **12255 ± 3251** | **n.a.** | **29000 ± 7162** | **7576 ± 947** | **n.a.** |  |
| **(mL/h)** | **14.4** | **26.5** |  | **24.7** | **12.5** |  |  |
| n.a.: not applicable n.c.: not calculated τ = 24h and t = 72h Racc: Accumulation ratio | | | | |  |  |  |

| 1. Study#3 | P14-03 (MAD for 21 days) |  |  |  | |  | |  | |  | |  | |  | | |  |
| --- | --- | --- | --- | --- | --- | --- | --- | --- | --- | --- | --- | --- | --- | --- | --- | --- | --- |
|  |  |  |  |  | |  | |  | |  | |  | |  | | |  |
| **Dose level** | **Sex** |  | **C_max_** | | **t_max_^*^** | | **t_1/2_** | **AUC_0-t_** | **R_acc_** | | **R_acc_** | |  | |  |  |  |
| **(µg)** |  |  | **(pg/mL)** | | **(h)** | | **(h)** | **(h*pg/mL)** | **C_max_** | | **AUC_0-24_** | |  | | |  |  |
| **30** | **Male** | **N** | 9 | | 9 | | 5 | 9 | 9 | | 9 | |  | | |  |  |
|  |  | **Mean** | 135.85 | | 4 | | NC | 8340.76 | 1.44 | | 1.98 | |  | | |  |  |
|  |  | **CV%** | 25.3 | | 1.5-8.0 | | NC | 29 | 25.1 | | 17.2 | |  | | |  |  |
|  |  | **GM** | 132.04 | | - | | NC | 8035.33 | 1.4 | | 1.95 | |  | | |  |  |
| **60** | **Male** | **N** | 9 | | 9 | | 7 | 9 | 9 | | 9 | |  | | |  |  |
|  |  | **Mean** | 268.21 | | 2.5 | | 87.7 | 14297.7 | 1.74 | | 2.08 | |  | | |  |  |
|  |  | **CV%** | 15.3 | | 2.0-6.0 | | 71 | 23.2 | 24.8 | | 25.7 | |  | | |  |  |
|  |  | **GM** | 265.45 | | - | | 70.4 | 13969.33 | 1.69 | | 2.02 | |  | | |  |  |
| **90** | **Male** | **N** | 9 | | 9 | | 6 | 9 | 9 | | 9 | |  | | |  |  |
|  |  | **Mean** | 353.85 | | 2.5 | | 208.4 | 20476.15 | 1.62 | | 2.09 | |  | | |  |  |
|  |  | **CV%** | 13.9 | | 1.5-6.0 | | 141.8 | 17.5 | 23.7 | | 23.5 | |  | | |  |  |
|  |  | **GM** | 350.37 | | - | | 123.8 | 20225.16 | 1.58 | | 2.04 | |  | | |  |  |
| **60** | **Female** | **N** | 9 | | 9 | | 9 | 9 | 9 | | 9 | |  | | |  |  |
|  |  | **Mean** | 303.36 | | 6 | | 139.1 | 24104.1 | 2.05 | | 2.69 | |  | | |  |  |
|  |  | **CV%** | 14.1 | | 1.5-8.0 | | 33.9 | 20.4 | 30.1 | | 20.4 | |  | | |  |  |
|  |  | **GM** | 300.77 | | - | | 132.9 | 23710.41 | 1.96 | | 2.65 | |  | | |  |  |
| * Median and Min-Max instead of Mean and CV% | | | |  | |  | |  | |  | |  | |  | | |  |
| NC: Not calculable as N < 2/3 | | |  |  | |  | |  | |  | |  | |  | | |  |
|  |  |  |  |  | |  | |  | |  | |  | |  | | |  |

Table S04: Demographic characteristics of Subjects in phase 1 to 2 (+ Alcohol Use in studies with AUD)

|  |  |
| --- | --- |
| **Figure S01: Gene reporter assay using CHO-DUKX-MRE/CRE-Luc cells** **stably expressing hH3R.**  Left: inhibition by pitolisant and BP1.3656B of the forskolin-stimulated cAMP production.  Right: reversal by pitolisant and BP1.3656B on the histamine-induced decrease of the forskolin-stimulated cAMP production.  ALU: arbitrary luminescence unit | |

|  |  |
| --- | --- |
| **Figure S02: β-arrestin recruitment assay on CHO-K1 cells stably expressing the human hH3R.**  Left: Stimulating effect of histamine whereas pitolisant and BP1.3656B alone are inactive.  Right: Reversal by pitolisant and BP1.3656B of histamine-induced β-arrestin recruitment. | |

|  |
| --- |
| **Figure S03: Pharmacokinetics of BP1.3656B in male Wistar rats (3 mg/kg, p.o.)**  Mean ± SEM of 4 rats. |

|  |  |
| --- | --- |
| **Figure S04: Effect of BP1.3656B on t-MeHA level in the brain.**  Left: Vehicle, BP1.3656B (0.003, 0.01, 0.03, 0.1, 0.3 and 1 mg/kg) or ciproxifan (1 mg/kg) were administered p.o. 90 minutes before sacrifice. t-MeHA whole brain levels are expressed in percent increase as compared to levels in control mice (254 ± 11 ng/g). Mean ± SEM of 20 female OF1 mice.  Right: Vehicle, BP1.3656B (0.03, 0.1, 0.3 and 1 mg/kg) or ciproxifan (3 mg/kg) were administered p.o. 90 minutes before sacrifice. t-MeHA brain cortex levels are expressed in ng/g of brain cortex. Mean ± SEM of 12-18 male Wistar rats. | |

|  |
| --- |
| **Figure S05: Effect of BP1.3656B on t-MeHA level in the brain of male OF1 mice.**  Reversal of BP1.3656B central effects on t-MeHA level by the histamine H_3_-receptor agonist imetit. Vehicle, ciproxifan (3 mg//kg), BP1.3656B (0.02 mg/kg), imetit (1 or 10 mg/kg) or BP1.3656B (0.02 mg/kg) combined with increasing doses of imetit (1, 3 and 10 mg/kg) were administered orally 90 min before sacrifice. t-MeHA levels are expressed in ng of t-MeHA/g of tissue. Mean ± SEM of 12-18 mice. Statistics: 1-way ANOVA F_(7,112)_=87.42 *P* < 0.001, followed by a PLSD Tukey test ** *P* < 0.01, *** *P* < 0.0001 *versus* control mice and ### *P* < 0.001 *versus* BP1.3656B 0.02 mg/kg alone. |

| **** |
| --- |
| **Figure S06: Effects of BP1.3656B (0. 1 to 0.3 mg/kg) on catecholamine turnover indices in the mouse cortex determined 90 minutes post oral administration.**  The following ratios are presented: DOPAC/DA (dihydroxyphenyl acetic acid/dopamine), HVA/DA (homovanilic acid/dopamine), MHPG/NA (4-hydroxy-3-methoxy-phenylglycol/noradrenaline) and 5-HIAA/5-HT (5-hydroxyindolacetic acid/serotonin). Mean ± SEM of 8 male C57BL/6J mice. Statistics: 1-way ANOVA indicated non-significant effects. |

| **** |
| --- |
| **Figure S07: Effects of BP1.3656B on ethanol 10% consumption in the drinking in the dark test in male C57BL/6J mice.**  Vehicle or BP1.3656B (0.3 mg/kg) were administered i.p. 30 minutes before the 2-hour test. Mean ± SEM of 12-20 mice. Statistics: paired Student t-test $ *P* < 0.05 *versus* ethanol / vehicle group. |

|  |
| --- |
| **Figure S08: Effects of BP1.3656B on anxiety behaviour in the elevated plus maze test after 24 hours of ethanol withdrawal following a 24 to 29-day two-bottle free choice drinking in male C57BL/6J mice.**  Vehicle or BP1.3656B (0.3 mg/kg) were administered i.p. 20 hours and 30 minutes before EPM test. The open bar represents the only-water group and hatched bars represent groups with one ethanol bottle available during the two-bottle choice procedure. Mean ± SEM of 5-15 mice. Statistics: paired Student t-test ** *P* < 0.01 *versus* no-withdrawal group (no-WD) and paired Student t-test $$ *P* < 0.01 *versus* ethanol-withdrawal group (ethanol-WD) treated with vehicle. |
